# The spatial distribution of GPCR and Gβγ activity across a cell dictates PIP3 dynamics

**DOI:** 10.1038/s41598-023-29639-0

**Published:** 2023-02-16

**Authors:** Dhanushan Wijayaratna, Kasun Ratnayake, Sithurandi Ubeysinghe, Dinesh Kankanamge, Mithila Tennakoon, Ajith Karunarathne

**Affiliations:** 1 Department of Chemistry and Biochemistry, The University of Toledo, Toledo, OH 43606 USA; 2 Department of Chemistry, Saint Louis University, 3501 Laclede Avenue, Saint Louis, MO 63103 USA; 3 Department of Anesthesiology, Washington University School of Medicine, Saint Louis, MO 63110 USA

**Keywords:** Molecular biology, Systems biology, Cell biology, Cell signalling, Cellular imaging, Biochemistry, DNA, Biological techniques, Imaging, Microscopy, Optogenetics, Sensors and probes

## Abstract

Phosphatidylinositol (3,4,5) trisphosphate (PIP3) is a plasma membrane-bound signaling phospholipid involved in many cellular signaling pathways that control crucial cellular processes and behaviors, including cytoskeleton remodeling, metabolism, chemotaxis, and apoptosis. Therefore, defective PIP3 signaling is implicated in various diseases, including cancer, diabetes, obesity, and cardiovascular diseases. Upon activation by G protein-coupled receptors (GPCRs) or receptor tyrosine kinases (RTKs), phosphoinositide-3-kinases (PI3Ks) phosphorylate phosphatidylinositol (4,5) bisphosphate (PIP2), generating PIP3. Though the mechanisms are unclear, PIP3 produced upon GPCR activation attenuates within minutes, indicating a tight temporal regulation. Our data show that subcellular redistributions of G proteins govern this PIP3 attenuation when GPCRs are activated globally, while localized GPCR activation induces sustained subcellular PIP3. Interestingly the observed PIP3 attenuation was Gγ subtype-dependent. Considering distinct cell-tissue-specific Gγ expression profiles, our findings not only demonstrate how the GPCR-induced PIP3 response is regulated depending on the GPCR activity gradient across a cell, but also show how diversely cells respond to spatial and temporal variability of external stimuli.

## Introduction

The cell is the ultimate building block of living organisms, perceives the extracellular environment in the form of chemical or physical stimuli employing cell surface receptors and respond accordingly^1^. Stimuli can be either chemicals such as hormones, or odorants, or physical signals, including light^2^. Not only activated receptors initiate signaling at the plasma membrane, but they also broadcast signals to the cell interior through effector molecules, controlling a variety of physiological processes^2,3^.If cells receive excessive stimulation, several protective mechanisms safeguard cells against sustained and deleterious signaling^3^. One such mechanism is desensitization of G protein coupled receptors (GPCRs), in which activated receptors trigger mechanisms for their phosphorylation and subsequent internalization^4^ Another mechanism is signaling adaptation. During adaptation, signaling response is reduced to the pre-activation level despite continuous stimulation^3^. In an adapted system, only a stronger stimulus could elicit a response. Adaptive biological systems are found in species ranging from bacteria to eukaryotes. In eukaryotes, signaling adaptation is involved with many crucial physiological processes, including pain, vision, and olfaction^3,5^.

Upon GPCR activation, conformational changes in Gα induce the exchange of bound GDP to GTP, inducing Gβγ dissociation from GαqGTP. These dissociated G proteins subsequently activate effector proteins, initiating downstream cellular signaling^6^. Literature provides evidence for signaling responses mediated by GPCRs and G proteins to be adaptive or attenuating^2,3,7^. For instance, Ras activation, phosphoinositide production, Protein kinase B (PKB) activation, cAMP production, and calcium influx have been identified as GPCR-mediated processes exhibiting self-attenuating responses, while in the presence of continuous stimuli^3^. Additionally, transient reduction of plasma membrane PIP2 upon GPCR activation has been observed^7^, and we recently showed the mechanisms underlying this self-attenuation process^8^.

As major regulators of cell fate and pathogenesis, kinases and phosphatases play crucial roles in phosphoinositide signaling by regulating cellular phosphoinositide levels^9^. For example, dysregulated expression and compartmentalization of specific kinases in the proper balance of phosphorylation-dephosphorylation events in cells have been implicated in cancer^9^. By catalyzing the phosphorylation of PIP2, Phosphoinositide 3-kinases (PI3Ks) generates PIP3, a crucial signaling phospholipid^10–12^. So far, three classes of PI3Ks (class I, II, and III) have been identified^12^. Though there are three classes of PI3Ks with multiple isoforms in each class^12^, only class I_A_ PI3Kβ (p110β catalytic and p85 regulatory) and I_B_ PI3Kγ (p110γ catalytic and p101 regulatory) are activated by Gβγ released upon GPCR stimulation^13,14^. These catalytic-regulatory dimers are cytosolic and recruited to the plasma membrane by Gβγ generated upon GPCR activation^14–16^. PIP3 is involved in crucial cellular regulation processes such as phagocytosis, localization of protein kinases, regulation of GTPases^17^, and multiple physiological processes, including cell migration, tissue regeneration, and chemotaxis^18^. In addition to GPCRs, PIP3 is also generated upon activation of other signaling pathways, including receptor tyrosine kinases such as insulin receptors^19^. Phospholipids act as docking sites for intracellular signaling proteins by recruiting multiple effector proteins to the plasma membrane. PIP3 recruits effectors to the plasma membrane mainly through interactions with Pleckstrin homology (PH) domains. PI3K-induced PIP3 generation leads to Akt and mTOR pathway activation, reducing apoptosis and enhancing cell proliferation^20^. Hence, many viral GPCRs activate PI3Ks to produce PIP3, benefiting the viral life cycle. Furthermore, viral GPCRs are constitutively active, and thus PI3Ks could remain active indefinitely^21^. Therefore, PI3K/Akt/mTOR signaling becomes tumorigenic and allows viral genome replication^22^.

We and others have shown that Gi/o-coupled GPCRs generate a significantly higher amount of Gβγ, which activates several effectors including PI3Kγ^12,15,23–25^. We have also demonstrated that the efficacy of PIP3 generation is dependent on the type of Gγ in the Gβγ dimer, and Gγ with high membrane affinity is a requirement of effective PIP3 generation^23^. PI3K/Akt pathway is tightly regulated in healthy cells^10^. Previously, it has been shown that GPCR activation-induced PIP3 exhibits attenuation within minutes upon a continuously applied stimulus^25–29^. Interestingly, when GPCRs are activated in a localized region of a cell, we observed that the resultant PIP3 response was attenuation-resistant. However, a molecular explanation for these distinct PIP3 responses in eukaryotic cells is lacking.

Here, using live-cell imaging, subcellular optogenetic GPCR, G protein activation, and single-cell analysis utilizing multiple biosensors, we examined the dynamic regulation of GPCR-activation-induced PIP3 in live cells. Our results show the molecular reasoning behind the distinct regulatory mechanisms that allow cells to trigger self-attenuating PIP3 generation when GPCRs are activated symmetrically and globally across a cell, while producing sustained localized PIP3 upon asymmetric and localized GPCR activation.

## Results and discussion

Please see Supplementary Figs. S8 and S9 for the monochromatic version of images in the main and supplementary figures.

### Gi/o-coupled GPCRs induce a self-attenuating PIP3 production

**Figure 1:**
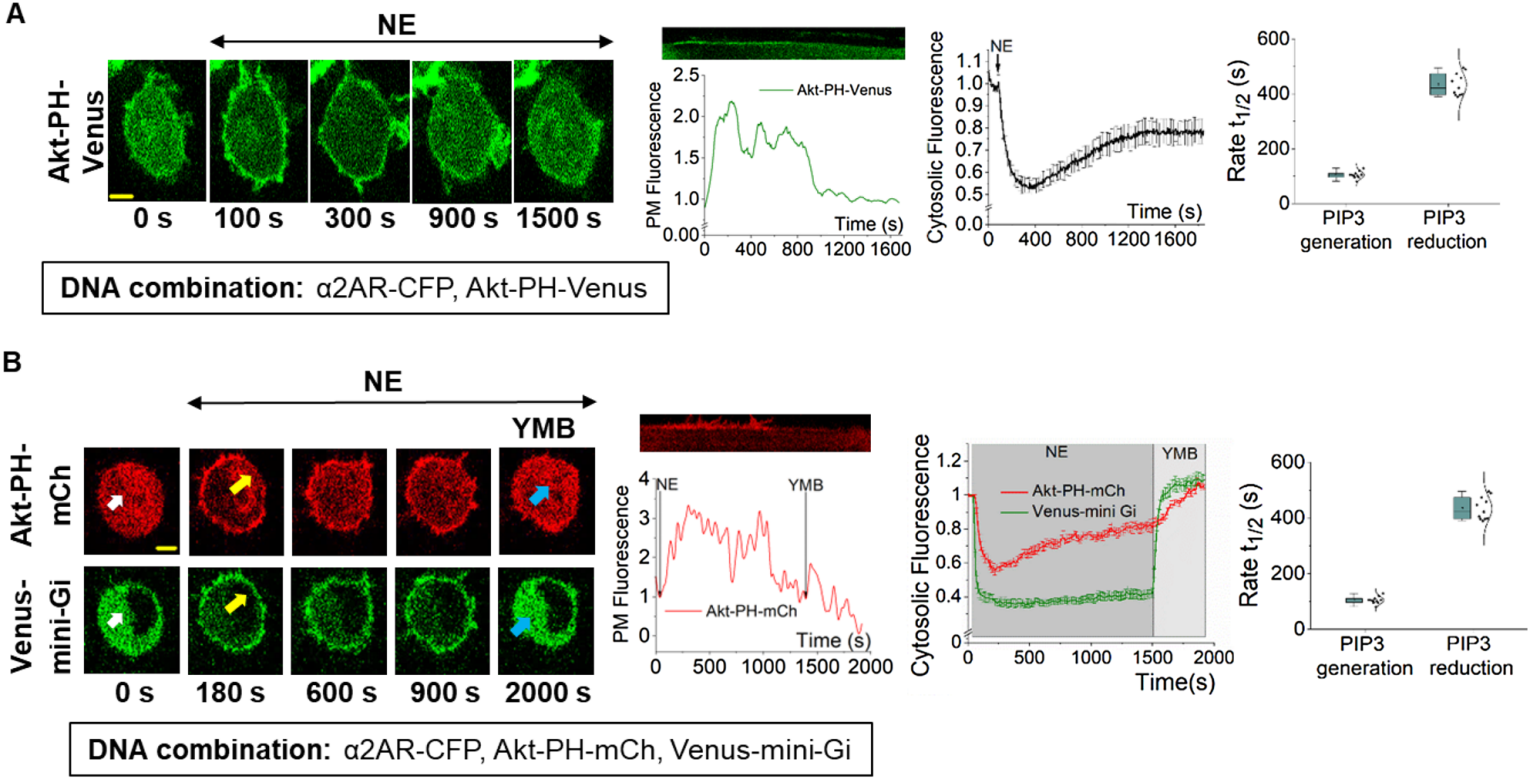
While GPCR and G proteins stay active, Gi/o-GPCR induced PIP3 subsides. (**A**) RAW264.7 cells exhibited robust PIP3 production upon α2AR activation, which shows subsequent significant attenuation. Images: RAW264.7 cells expressing Akt-PH-Venus (PIP3 sensor) and α2AR-CFP (not shown). Confocal time-lapse imaging of PIP3 sensor dynamics was performed using 515 nm excitation. α2AR was activated using 100 µM NE at 1 min. Images show PIP3 sensor translocation from the cytosol to the PM upon α2AR activation, which peaks at 300 s, and significantly reverses back to the cytosol in 5–10 min. The corresponding plot shows PIP3 sensor dynamics in the cytosol of the cells. The whisker box plot shows the half-time (t_1/2_) of PIP3 generation and attenuation (n = 13). (**B**) RAW264.7 cells expressing α2AR-CFP, Akt-PH-mCh, and Venus-mini-Gi exhibited simultaneous mini-Gi PM-recruitment and PIP3 production (yellow arrows) upon α2AR activation. 100 µM NE was added at 1 min to activate α2AR. Mini-Gi stayed recruited to the PM, while PIP3 showed attenuation. Upon addition of α2AR inhibitor yohimbine (50 µM), both mini-Gi and PIP3 sensors showed complete reversal to the cytosol (blue arrows). Cells were imaged using 515 nm (to monitor mini-Gi), and 594 nm (to capture the PIP3 sensor) excitation. The corresponding plot shows the mini-Gi (green) and PIP3 (red) sensor dynamics in the cytosol of the cells. The whisker box plot shows the half-time (t_1/2_) of PIP3 generation and attenuation rates (n = 11). Kymographs show PIP3 levels on the plasma membrane. Average curves were plotted using cells from ≥ 3 independent experiments. ‘n’ denotes the number of cells. Error bars represent SEM (standard error). The scale bar = 5 µm. *GPCR* G protein-coupled receptor, *PIP3* Phosphatidylinositol 3,4,5 triphosphate, *α2AR* Alpha-2-adrenergic receptor, *NE* Norepinephrine, *CFP* cyan fluorescence protein, *mCh* mCherry, *PM* plasma membrane.

Gi/o-coupled GPCR activation stimulates PI3Kγ at the plasma membrane leading to PIP3 generation^16,17,23^. We activated Gi/o-coupled alpha 2 adrenergic receptors (α2AR) using 100 μM norepinephrine and examined the PIP3 generation in RAW264.7 mouse macrophage cells. PIP3 response was observed using the PIP3 sensor, Akt-PH-Venus, which is cytosolic in the absence of PIP3 and translocates to the plasma membrane upon PIP3 generation. We observed a robust PIP3 production with a half-time (t_1/2_) of = 109.78 ± 4.01 s (Fig. 1A, up to 300 s, and plots). Here, we measured PIP3 production using the reduction in cytosolic PIP3 sensor fluorescence due to its plasma membrane recruitment to avoid experimental artifacts because PIP3 causes cell changes, introducing artifacts to membrane fluorescence measurement. Further confirming that cytosolic fluorescence reflects PIP3 dynamics at the plasma membrane, single-cell plasma membrane fluorescence measurements using kymographs also recapitulated the observed PIP3 dynamics. Interestingly, after PIP3 production reached the maximum, Akt-PH returned to the cytosol with a t_1/2_ of = 433.85 ± 12.13 s, indicating the reduction of PIP3 at the plasma membrane (Fig. 1A, 900 and 1500 s and plots). In a control experiment, cells failed to produce PIP3 in the absence of α2AR expression (Supplementary Fig. S5).

Since we did not remove the α2AR agonist, norepinephrine, we wanted to examine whether the observed PIP3 attenuation is a result of GPCR or G protein inactivation. GPCR-G protein activation-induced cellular processes are usually terminated upon GPCR deactivation due to agonist removal, exposing the receptor to antagonists or GPCR desensitization and internalization upon their phosphorylation^30,31^. Therefore, using a fluorescently-tagged mini-G protein, we examined whether a reduction of cell surface concentration of active α2AR is responsible for the observed PIP3 reduction. Mini-G proteins are cytosolic sensors recruited to active GPCRs upon agonist addition^32^. Since α2AR is Gi/o-coupled, we expressed Venus-mini-Gi and Akt-PH-mCherry (Akt-PH-mCh) to monitor the status of the receptor and PIP3 response simultaneously in the same cell. Before activation, both sensors were cytosolic (Fig. 1B, 0 min- white arrows). Norepinephrine addition induced simultaneous PIP3 generation and mini-Gi recruitment to the plasma membrane (Fig. 1B- yellow arrows). Continuous monitoring showed that PIP3 gradually and significantly disappeared with a t_1/2_ of = 426.39 ± 19.54 s (Fig. 1B-red plot). However, mini-Gi stayed on the plasma membrane, indicating active-state GPCR (Fig. 1B- green plot). Therefore, the observed PIP3 disappearance meets the criteria of a partial adaptation because, during the process, GPCRs remained active. The addition of α2AR antagonist, yohimbine (50 µM) deactivated α2ARs, resulting in a complete reverse translocation of mini-Gi and the PIP3 sensor to the cytosol (Fig. 1B- blue arrows). These data collectively show that the diminished GPCR activity is not a significant factor in the observed partial adaptation of PIP3.

### PIP3 attenuation is not a result of the substrate, PIP2 or product, PIP3 depletion by phosphatases

**Figure 2:**
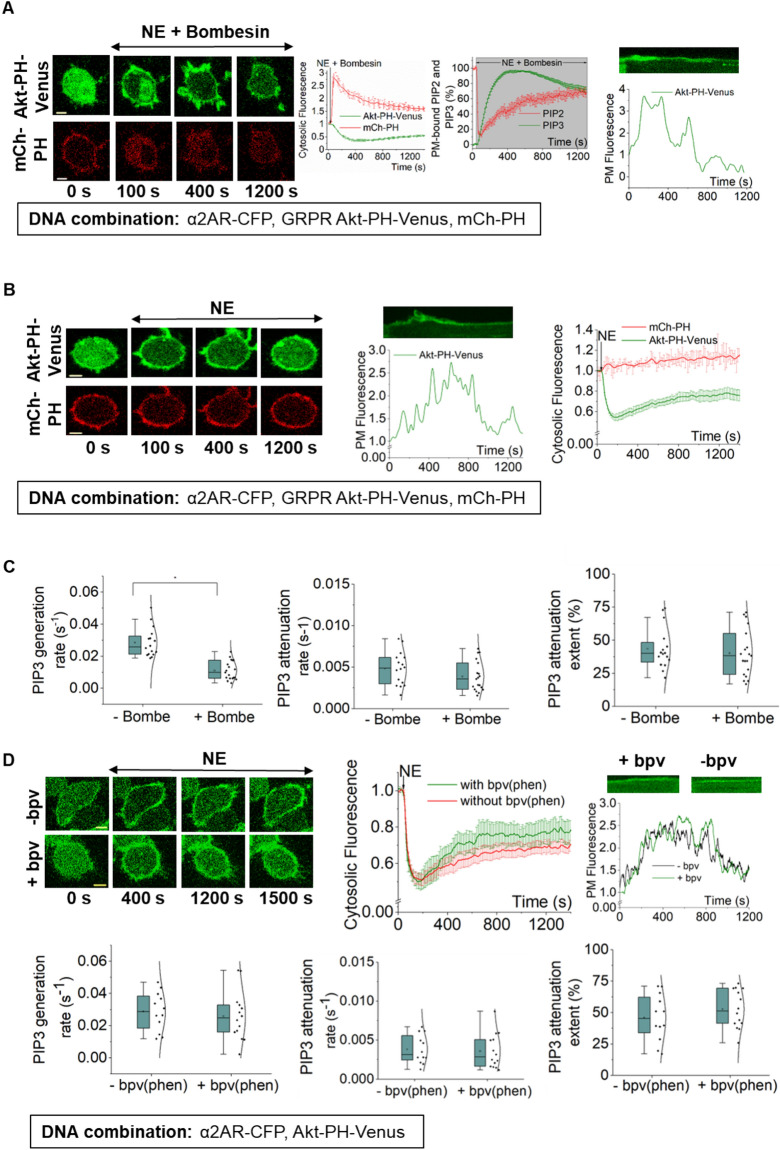
PIP3 attenuation is not a result of substrate (PIP2) depletion. (**A**) RAW264.7 cells expressing α2AR-CFP, GRPR, Akt-PH-Venus, and mCh-PH exhibited concurrent PIP2 hydrolysis—PIP3 production, and PIP3 attenuation upon simultaneous GRPR and α2AR activation at 1 min using 100 µM NE and 1 µM bombesin, respectively. Cells were imaged using 515 nm (for PIP3 sensor), and 594 nm (for PIP2 sensor). The corresponding plot shows the dynamics of PIP2 hydrolysis (red) and PIP3 production and attenuation (green). The plot compares the normalized PIP2 (red) and PIP3 (green) sensor dynamics at the PM. The two plots show that the changes in the sensor fluorescence in cytosol or the PM can be used to capture the dynamics of PIP2 and PIP3 at the PM (n = 19). (**B**) RAW264.7 cells expressing α2AR-CFP, GRPR, Akt-PH-Venus, and mCh-PH exhibited PIP3 production and attenuation upon α2AR activation (at 1 min), while PIP2 at the PM stayed intact. The corresponding plot shows the dynamics of PIP3 (green) and PIP2 (red) (n = 16). (**C**) The whisker box plots show PIP3 generation rates, attenuation rates, and attenuation extents for A and B experiments indicating the influence of PIP2 hydrolysis on PIP3. PIP3 generation rate in cells with PIP2 hydrolysis (+ Bombesin) was significantly lower compared to cells without PIP2 hydrolysis (-Bombesin). However, the rate and extent of PIP3 attenuation under both conditions showed no significant difference. (**D**) RAW264.7 cells expressing α2AR-CFP and Akt-PH-Venus exhibited PIP3 production and attenuation upon α2AR activation in the presence or absence of phosphatase inhibitor bpV(phen) (5 µM). The corresponding plot shows the dynamics of PIP3 production and attenuation in the presence of bpV(phen) (green) (n = 14), and in the absence of bpV(phen) (red) (n = 12). The whisker box plots show PIP3 generation rates, attenuation rates, and attenuation extents in the presence and absence of bpV(phen). The generation and attenuation rates and attenuation extents showed no significant difference under both conditions. Kymographs show PIP3 levels on the plasma membrane. Statistical comparisons were performed using One-way-ANOVA (p < 0.05). Average curves were plotted using cells from ≥ 3 independent experiments. ‘n’ denotes the number of cells’ data used to plot the average curve. The error bars represent SEM. Scale bar = 5 µm. *PIP2* phosphatidylinositol 4,5-bisphosphate, *GRPR* gastrin-releasing peptide receptor.

Active PLCβ hydrolyzes PIP2, generating inositol triphosphate (IP3) and diacylglycerol (DAG), and PIP2 is the substrate for PI3K to generate PIP3^33^. Though GαqGTP is the highly efficient PLCβ activator, Gβγ also stimulates PLCβ and induces PIP2 hydrolysis^34^. We have shown that PIP2 hydrolysis induced by Gβγ alone is modest compared to the robust hydrolysis induced by GαqGTP^8^. Since α2AR is a Gi/o-GPCR, upon activation, Gβγ should be the only available PLCβ activator. Therefore, we examined whether depletion of plasma membrane-bound PIP2 by Gβγ retards PIP3 generation. We coexpressed α2AR, GRPR (Gq-GPCR), mCh-PH (PIP2 sensor), and Akt-PH-Venus (PIP3 sensor) in RAW 264.7 cells. Upon addition of 100 μM norepinephrine and 1 μM bombesin simultaneously, both α2AR and GRPR were activated, and PIP2 hydrolysis was observed (Fig. 2A). Interestingly, despite the robust PIP2 hydrolysis, we also observed a simultaneous PIP3 production and its subsequent attenuation. This showed that, even with significantly reduced PIP2 availability at the plasma membrane (Fig. 2A, PM-bound PIP2, and PIP3 plot), PI3K could still generate PIP3. We also examined PIP2 dynamics at the plasma membrane during Gi/o-GPCR-induced PIP3 level adapts. We coexpressed the same DNA combination as above and activated only α2AR. PIP2 sensor fluorescence at the plasma membrane remained unchanged during the PIP3 generation and its attenuation (Fig. 2B). These two conditions, in the presence (Fig. 2A) and absence (Fig. 2B) of Gq-GPCR activity, have created two distinct PIP2 availabilities at the plasma membrane for PI3Ks to generate PIP3. The PIP3 generation was significantly slower (⁓2.5 fold) in the presence of Gq-GPCR activation compared to that in the absence of Gq-GPCR activation (0.0111 vs. 0.02858 s^-1^) (Fig. 2C—one-way ANOVA: *F*_1, 33_ = 47.05388, *p* = 7.87 × 10^–8^, Supplementary Table S2 A and B). This can be explained assuming the distinct concentrations of PIP2 available for PI3K, where PIP3 generation is impeded under low PIP2 abundance. However, the PIP3 attenuation rates or extents with and without Gq-GPCR activation showed no significant difference (attenuation rate: one-way ANOVA: *F*_1, 33_ = 2.36795, *p* = 0.133, attenuation extent: one-way ANOVA: *F*_1, 33_ = 0.32997, *p* = 0.56957) (Fig. 2C, Supplementary Table S3 A and B, Supplementary Table S4 A and B). This data suggests that while PIP2 availability at the plasma membrane could affect the magnitude and kinetics of PIP3 generation, it does not influence PIP3 attenuation. There have been discussions about the specificity of the PIP2 sensor and its ability to interact with PIP3^17^. Nevertheless, our data also show that even with strong PIP3 production at the plasma membrane, the PIP2 sensor stayed cytosolic after PIP2 hydrolysis (Fig. 2A, 100 and 400 s, and plots). This clearly shows that under the experimental conditions we employed, the PIP2 sensor is sufficiently specific for PIP2.

Cellular phosphatases such as PTEN reduce cellular PIP3 levels and regulate downstream signaling^35–38^. However, PTEN is not the only PIP3 phosphatase in cells. RNA seq data of RAW264.7 cells show a significantly higher expression of several phosphatases, including PTEN, Inpp5d, and Inpp5b (Supplementary Fig. S3). Therefore, to examine whether phosphatases regulate PIP3 attenuation, we employed the phosphatase inhibitor, bpV(phen), which has been shown to inhibit most phosphatases, including the above^39,40^. Cells expressing α2AR and Akt-PH-Venus were pre-incubated with 5 µM inhibitor for 30 min, and norepinephrine was added to activate α2AR. Upon activation, both control and phosphatase-inhibited cells showed PIP3 production and subsequent attenuation (Fig. 2D). The PIP3 generation rates (one-way ANOVA: *F*_1,__24_ = 0.29921, *p* = 0.58943, Supplementary Table S5A and B), PIP3 attenuation rates (one-way ANOVA: *F*_1,__24_ = 0.08259, *p* = 0.77629, Supplementary Table S6A and B), and the PIP3 attenuation extents (one-way ANOVA: *F*_1,__24_ = 1.04804, *p* = 0.31617, Supplementary Table S7A and B) do not show a significant difference. This showed that neither PIP2 depletion nor phosphatase activity influenced PIP3 attenuation.

### Plasma membrane residency of Gβγ and the kinetics of the PIP3 attenuation

**Figure 3:**
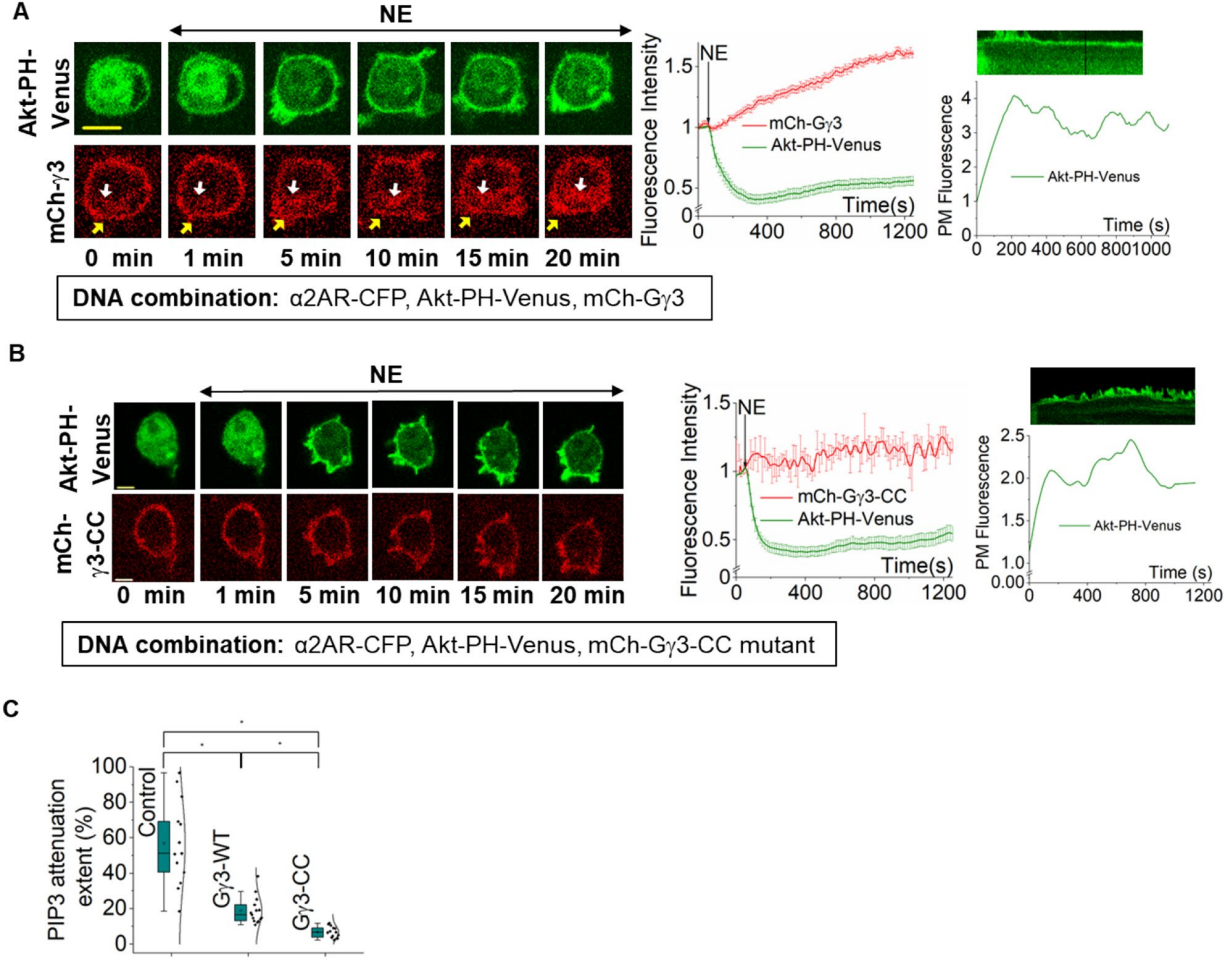
Gβγ and PIP3 attenuation (**A**) RAW264.7 cells expressing α2AR-CFP, Akt-PH-Venus and mCh-Gγ3 exhibited simultaneous mCh-Gγ3 translocation and PIP3 production upon α2AR activation (at 1 min). The translocated mCherry-Gγ3 stayed at IMs (white arrows), while PIP3 level reached an equilibrium after attenuation. In addition to 515 nm excitation for YFP, 594 nm excitation was used to capture mCherry. The corresponding plot shows mCh-Gγ3 (red) and PIP3 (green) dynamics in the cytosol of the cells (n = 20). (**B**) RAW264.7 cells expressing α2AR-CFP, Akt-PH-Venus and mCh-Gγ3-CC mutant exhibited PIP3 production upon α2AR activation. As expected, the images and the plot show that translocation deficient Gγ mutant (Gγ3-CC) remained at the PM despite receptor activation. PIP3 showed no significant attenuation (n = 16). (**C**) The whisker box plots show the extents of % PIP3 attenuation in RAW264.7 cells with endogenous Gβγ (Control), or Gγ3 WT, or Gγ3-CC mutant. Extent of PIP3 attenuation was quantified using the increase in the mean cytosolic fluorescence due to PIP3 attenuation. The observed % attenuations were significantly different from each other (p < 0.05). Kymographs show PIP3 levels on the plasma membrane. Averages were plotted using cells from n ≥ 3 independent experiments. ‘n’ denotes the number of cells’ data used to plot the average curve. The error bars represent SEM (standard error of mean). The scale bar = 5 µm. *IMs* internal membranes.

The glycerolipid group of Phosphatidylinositol is plasma membrane-bound, and therefore, to phosphorylate the substrate PIP2, PI3K subunits are recruited to the plasma membrane by plasma membrane-bound Gβγ^13^. Thus, Gβγ concentration at the plasma membrane should be a critical determinant of PIP3 production. Previously, it has been shown that Gβγ subunits generated upon GPCR activation at the plasma membrane reversibly translocate to internal membranes (IMs), including endoplasmic reticulum (ER) and Golgi, in a Gγ-dependent manner^41,42^, and high membrane affinity Gγ types are translocation deficient^23^. Our previous work shows that PIP3 production upon GPCR activation requires the Gβγ composed of high membrane-affinity Gγ types^23^. Gγ3 shows the highest membrane affinity out of the 12 subtypes. Its expression facilitates the PIP3 production in HeLa cells, which typically do not show a significant PIP3 production upon GPCR activation due to the lack of high membrane-affinity Gγ expression at the endogenous level^23^. High membrane affinity Gβγ have the advantage of primarily residing at the plasma membrane even after being released from the heterotrimer^23^. Therefore, we hypothesized that the plasma membrane residency of Gβγ subunits and their translocation away from the plasma membrane regulates the observed PIP3 response attenuation. To examine whether Gβγ translocation and PIP3 response attenuation is related, we employed RAW 264.7 cells coexpressing α2AR-CFP, Akt-PH-Venus, and mCh-Gγ3. Activation of α2AR induced a robust PIP3 generation (Fig. 3A, green plot). As expected, Gγ3 showed slow and steady translocation indicated by mCherry fluorescence loss at the plasma membrane and gradual increase in the cell interior (Fig. 3C, red plot). Next, we analyzed the dynamics of Gγ3 translocation and PIP3 generation—attenuation. At the 5-min mark, the majority of Gγ3 (~ 89.18 ± 3.41%) still stayed plasma membrane-bound (Fig. 3A-yellow arrows, Supplementary Fig. S1 and Supplementary Table S1), while PIP3 generation reached the maximum (Fig. 3A, 5 min, and plot). Even at the 20-min mark, ~ 65.039 ± 2.68% of Gγ3 stayed plasma membrane-bound (Supplementary Fig. S1 and Supplementary Table S1), while PIP3 reduction had reached a steady state. Considering Gβγ is responsible for PIP3 generation, this data suggested that the percent PIP3 that remains at the plasma membrane is proportional to the Gβγ concentration. The extent of PIP3 response attenuation in WT-RAW264.7 cells (56.801 ± 6.61%) is ⁓threefold higher than that of the cells expressing Gγ3 (18.725 ± 1.93%) (Fig. 3E, Supplementary Table S8A). RNA seq data shows that RAW264.7 cells prominently express four major Gγ types at endogenous conditions, Gγ2 (36%), Gγ5 (14%), Gγ9 (17%), and Gγ12 (27%) (Supplementary Fig. S2 and Supplementary Table S9). Assuming that mRNA levels are proportional to protein expression, we can estimate the Gβγ concentration at the plasma membrane after its translocation reached the equilibrium in WT RAW264.7 cells since we have previously shown that Gγ2, 5, 9, and 12 translocate respectively 1.5, 3.8, 45, and 3.4 times faster than Gγ3^23^. For Gγ3-expressing cells, we assumed that the majority of Gγ (~ 90%) in Gβγ is Gγ3. Since we did not express either Gα or Gβ, we also assumed nearly similar heterotrimer concentrations in both cellular conditions. To examine whether expressing high membrane-affinity Gγ increases the plasma membrane-bound Gβγ at the steady state, we compared percent plasma membrane-bound Gβγ3 and Gβγ9 at the steady state of PIP3 attenuation (~ 20 min). Here, our data showed that Gγ3-expressing cells have ~ sixfold higher Gβγ concentration at the plasma membrane compared to that of the Gγ9-expressing cells (Supplementary Fig. S1 and Supplementary Table S1). Since expression of Gγ3 increases the plasma membrane-bound Gβγ concentration at the steady state, we compared the PIP3 reduction extent between Gγ3-expressing cells and endogenous Gβγ-expressing cells. Here, endogenous cells showed ~ threefold higher extent of PIP3 reduction (Fig. 3C- Control and Gγ3-WT, Supplementary Table S8A and B). To further examine whether the plasma membrane residency of Gβγ is linked to the PIP3 reduction, we employed a translocation-deficient Gγ3 mutant (Gγ3-CC). This mutant has an additional *Cys* residue at the C-terminus, resulting in double lipidation, significantly enhancing its membrane affinity beyond Gγ3-WT^8^. We have also shown that Gγ3-CC encodes for a functional Gγ^8^. We coexpressed α2AR-CFP, Akt-PH-Venus, and mCh-Gγ3-CC mutant in RAW 264.7 cells. Activation of α2AR induced PIP3 generation; however, the Gγ3-CC mutant showed no translocation (Fig. 3B). Mutant-expressing cells showed a nearly threefold decrease in the extent of PIP3 attenuation (6.759 ± 0.85%) compared to Gγ3-WT-expressing cells (one-way ANOVA: *F*_1, 33_ = 56.87, *p* = 1.13 × 10^–8^) (Fig. 3C, Supplementary Table S8A and B). Therefore, this data suggests that the continuous presence of active Gβγ on the plasma membrane in the presence of Gγ3-CC (Fig. 3B, red plot and cell images) allows for sustained PIP3 at the plasma membrane while preventing the PIP3 attenuation process (Fig. 3B, green plot and cell images). Further, membrane affinity differences in the above-considered Gγ types strongly suggest that the Gβγ loss from the plasma membrane due to translocation plays a crucial role in the dynamic attenuation of GPCR activation-induced PIP3.

To monitor the localization of PI3Kγ in PIP3 attenuation, we examined PI3Kγ dynamics alongside PIP3 in RAW264.7 cells. We expressed α2AR-CFP, Akt-PH-mCh, GFP-p110γ and untagged p101 in RAW264.7 cells. However, we observed that cells with high GFP-P110γ expression resulted in reduced attenuation of PIP3. Here, we believe that the effective PI3Kγ level is likely to be several-fold higher than that of the endogenous upon its transient expression. Therefore it is not surprising that this elevated PI3Kγ can more effectively produce PIP3 utilizing the remaining Gβγ at the plasma membrane. In cells with relatively low GFP-P110γ expression, we observed, P110γ translocation to the plasma membrane upon receptor activation and partially returning to the cytosol alongside the attenuation of PIP3 (Fig. S6), resembling the kinetics of PIP3 generation and attenuation. Further, in the same experiment, cells lacking GFP-P110γ expression consistently showed PIP3 production and attenuation (data not shown). These observations showed consistency with our hypothesis that Gβγ translocation away from the plasma membrane induces PIP3 attenuation.

### Reintroduction of Gβγ rescues attenuated PIP3

**Figure 4:**
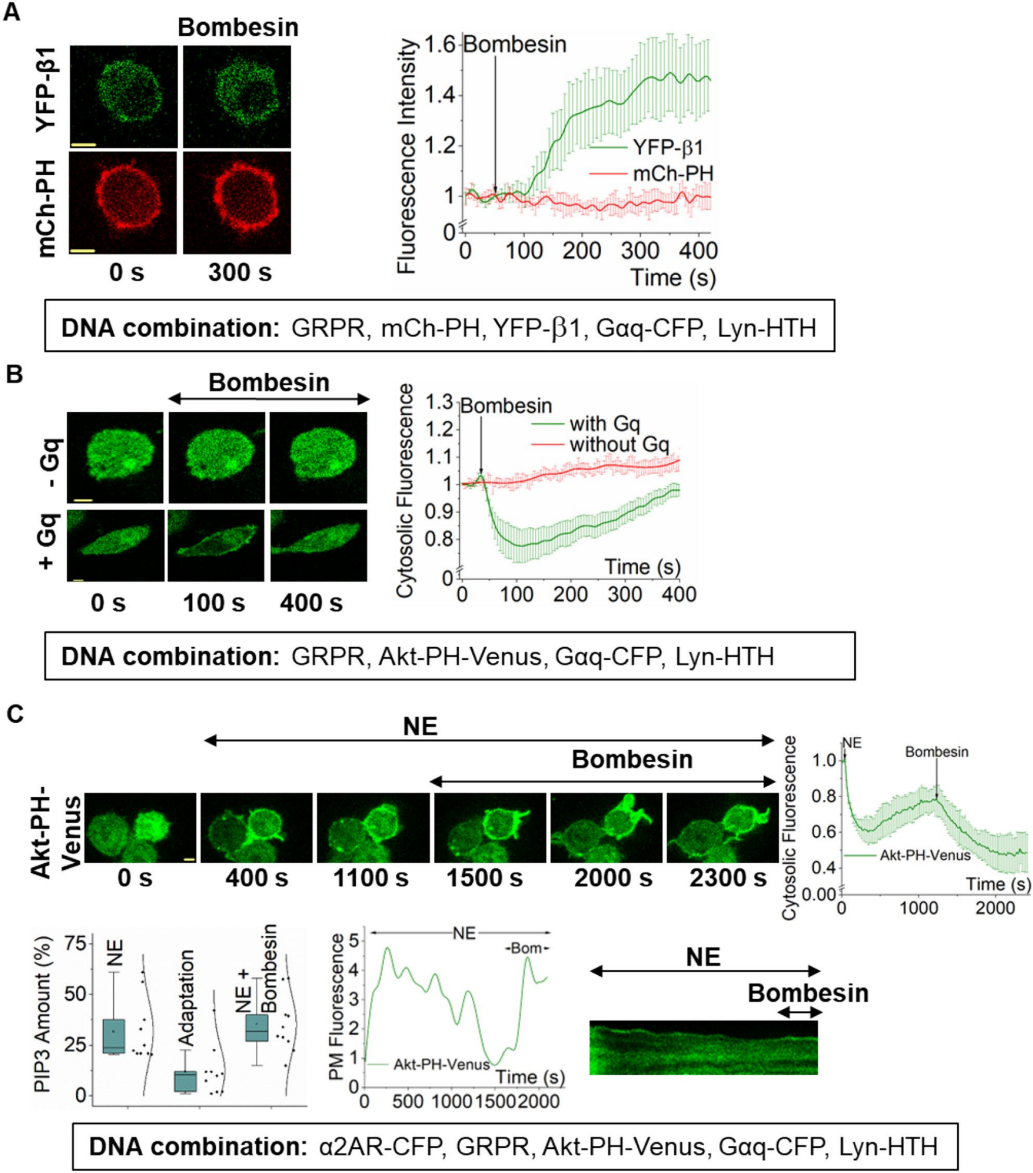
Despite the source, Gβγ entry induces PIP3 generation and loss results in attenuation. (**A**) RAW264.7 cells expressing GRPR, YFP-β1, mCh-PH, Gαq-CFP, and Lyn-HTH exhibited robust β1 translocation upon GRPR activation with bombesin (1 µM at 1 min) (n = 8). However, GRPR activation did not induce PIP2 hydrolysis due to the presence of Lyn-HTH. The plot shows the dynamics of β1 translocation (green) and PIP2 (red) in cells. (**B**) RAW264.7 cells expressing GRPR, Akt-PH-Venus, Gαq-CFP, and Lyn-HTH exhibited PIP3 production and attenuation upon GRPR activation with 1 µM bombesin (n = 8). However, control cells expressing only GRPR and Akt-PH-Venus did not show PIP3 production upon GRPR activation (n = 9). The plot shows PIP3 production and attenuation only in Gαq-expressing cells (green), however not in control cell lacking introduced Gαq. (**C**) RAW264.7 cells expressing α2AR-CFP, GRPR, Akt-PH-Venus, Gαq-CFP and Lyn-HTH exhibited PIP3 production and attenuation upon α2AR activation. After PIP3 attenuation, 1 µM bombesin was added (at 20 min) to activate GRPR. GRPR activation in these Gαq cells caused disruption of attenuation in the form of PIP3 increase. The plot shows the initial PIP3 generation and attenuation after α2AR activation, and subsequent GRPR-induced PIP3 regeneration in Gαq background (n = 10). Kymographs show PIP3 levels on the plasma membrane. Average curves were plotted using cells from ≥ 3 independent experiments. ‘n’ denotes the number of cells’ data used to plot the average curve. The error bars represent SEM (standard error of mean). The scale bar = 5 µm.

We next examined whether the attenuated PIP3 response can be rescued by injecting Gβγ into the system by activating Gq heterotrimers. RNAseq data shows that the endogenous Gαq expression in RAW264.7 cells is significantly lower compared to other Gα types, including Gαi/o (Supplementary Fig. S4 and Supplementary Table S11). Thus, it produces limited Gβγ upon Gq-GPCR activation, resulting in little to no PIP3 generation^43^. Since Gβγ is shared between different Gα types in a cell, we hypothesized that by expressing Gαq in RAW264.7 cells, we could increase Gq-GPCR activation-mediated Gβγ generation. We expressed GRPR (a Gq-coupled GPCR), Gαq-CFP, YFP-β1, and Lyn-HTH in RAW 264.7 cells. Since PLCβ-mediated PIP2 hydrolysis significantly impedes PIP3 production (Fig. 2A,C, Supplementary Table S2A and B), we employed plasma membrane-targeted HTH (Helix-Turn-Helix) domain of PLCβ3 (from Y847-E884) since it competes with PLCβ to interact with GαqGTP, and has been shown to inhibit PIP2 hydrolysis^44^. The data show that Lyn-HTH completely inhibited Gαq-induced PLCβ stimulation; however, it did not prevent Gβγ translocation, indicating unperturbed heterotrimer activation (Fig. 4A). To examine Gq-GPCR activation-mediated Gβγ can generate PIP3, we expressed GRPR, Gαq-CFP, Akt-PH-Venus, and Lyn-HTH in RAW 264.7 cells. As expected, a robust PIP3 generation and subsequent attenuation were observed in Gαq-expressing cells upon GRPR activation (Fig. 4B, + Gq). However, in the absence of Gαq expression, GRPR activation did not induce PIP3 production (Fig. 4B, -Gq). This data also ruled out the possibility of Gαi involvement in PIP3 generation or its attenuation. Since GRPR activation in Gαq expressing cells exhibited a robust Gβγ generation that is even sufficient to induce a significant PIP3 generation, we next examined whether the PIP3 attenuation observed after Gi/o-GPCR could be rescued by reintroducing Gβγ using Gq-GPCR pathway activation. We expressed α2AR-CFP, GRPR, Gαq-CFP, Akt-PH-Venus, and Lyn-HTH in RAW 264.7 cells. We first activated α2AR and observed PIP3 generation (Fig. 4C, 400 s, and plots). We continued imaging cells for 20 min till PIP3 at the plasma membrane was reduced to a constant level indicating the maximum attenuation (Fig. 4C, 1100 s, and plots). We then activated Gq-coupled GRPR using 1 μM bombesin. The attenuated PIP3 levels in cells were increased to the pre-attenuation level (compare Fig. 4C, 400 s, and 1500 s). This data suggests that Gβγ reintroduction can rescue the PIP3 attenuation, further suggesting that the loss of plasma membrane-bound Gβγ maybe the molecular reason behind PIP3 attenuation. Considering the lack of PIP3 attenuation observed in translocation-incompetent Gγ3 and translocation-deficient Gγ3 CC-mutant cells (Fig. 3A–C), this PIP3 rescue observed upon Gβγ injection clearly indicates the Gβγ involvement in the PIP3 attenuation.

### Localized optogenetic inhibition of active Gαi can disrupt the PIP3 attenuation process

**Figure 5:**
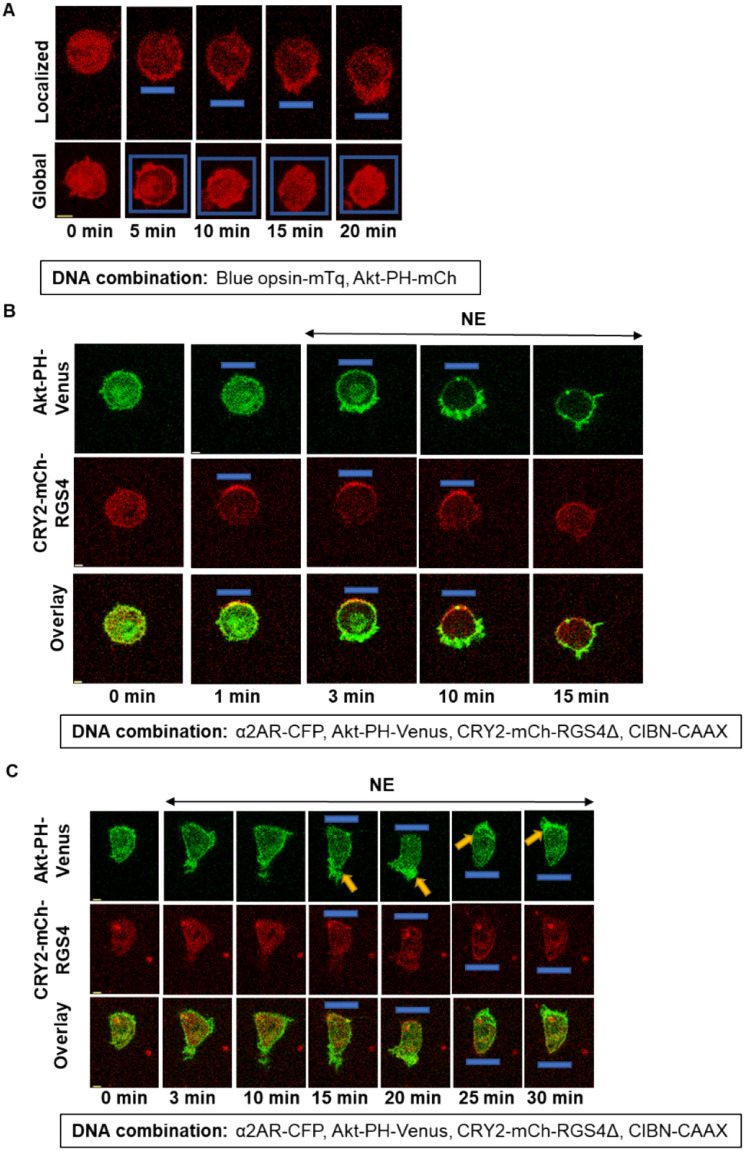
PIP3 generation upon localized GPCR-G protein activation is attenuation-resistant. (**A**) RAW264.7 cells expressing blue opsin-mTq and Akt-PH-mCh showed attenuation-resistant PIP3 upon localized activation, while global activation induced fast attenuating PIP3. Before activation cells were incubated with 1 µM 11-cis-retinal. Spatially confined blue light pulses (blue boxes) (1 Hz) were used to activate by blue light locally and globally, while confocal imaging of mCherry using 594 nm excitation. Cells activated with localized blue light produced non-attenuating PIP3 response. Cells also showed a directional migration towards the blue light. On the contrary, cells exposed to blue light globally initially showed a robust global PIP3 production. However, PIP3 in these cells showed the attenuation, similar to the attenuation observed upon activation of α2AR. (**B**) Localized acceleration of GTP hydrolysis on Gα in α2AR activated RAW264.7 cells also showed attenuation-resistant PIP3 generation. Cells expressing α2AR-CFP, Akt-PH-Venus, CRY2-mCh-RGS4Δ, and CIBN-CAAX showed localized PIP3 production when α2AR was activated in a cell after CRY2-mCh-RGS4Δ localized to one side of the cell. A localized blue light pulse was provided to recruit CRY2-mCh-RGS4Δ to one side of the cell. Once RGS4Δ is recruited, (indicated by mCherry fluorescence concentrating to one side of the cell), NE (100 µM) was added to the medium. This resulted in localized PIP3 at the opposite side to the blue pulse (yellow arrow). Cells were imaged using 515 nm (to capture PIP3 sensor), and 594 nm (to capture RGS4Δ). Although images show data from only one cell, experiments were conducted in multiple cells with > 3 independent experiments to test the reproducibility of the results. **(C)** PIP3-attenuated (post-GPCR activation) RAW264.7 cells show disruption of attenuation upon localized inhibition of  G protein activity. Cells expressed α2AR-CFP, Akt-PH-Venus, CRY2-mCh-RGS4Δ, and CIBN-CAAX. Initially α2AR was activated with 100 µM NE, and PIP3 was produced and significantly attenuated in 10 min. Upon blue light induced recruitment of CRY2-mCh-RGS4Δ to one side of the cell, PIP3 was produced at the opposite side (yellow arrow, 15 and 20 min), indicating attenuation disruption. When the opposite side of the same cell was exposed to blue light (yellow arrow, 25 and 30 min), PIP3 was produced at the opposite side of localized RGS4Δ. Experiments were conducted in multiple cells with > 3 independent experiments to test the reproducibility of the results. The blue box indicates the blue light. The scale bar = 5 µm. *mTq* mTurquoise, *FRAPPA* fluorescence recovery after photo-bleaching and photo-activation, *RGS4* regulator of G protein signaling 4.

Localized optical activation of a Gi/o-coupled GPCR, blue opsin, shows a sustained and attenuation -resistant localized PIP3 generation at the leading edge in migrating RAW264.7 cells (Fig. 5A- Localized)^45^. Here we used cells expressing blue opsin-mTurquoise and Akt-PH-mCh. After the addition of 1 µM 11-*cis*-retinal (to generate optically activatable blue opsin), and while imaging the cells for mCherry, we exposed a confined plasma membrane region of a cell to 445 nm blue light by exposing an adjacent area to rectangular-shaped blue light pulse delivered at 1 Hz. (Fig. 5A-Localized- Rectangular blue box). The opsin activation induced cell migration towards the localized blue light, which was steered to manage the migration direction. The cells showed a sustained localized PIP3. When a cell was exposed to blue light globally, although the PIP3 generation was observed, the PIP3 response attenuated quickly (Fig. 5A- Global). Since the asymmetric GPCR activation-mediated PIP3 production was attenuation resistant, we examined whether breaking the signaling symmetry in a PIP3- attenuated cell could also recover the cell from the PIP3 attenuation. Here we employed an optogenetic GTPase engineered using a truncated version of the Regulator of G protein signaling 4 domain (RGS4Δ) that has been used to optically inhibit G protein activity in cells^46^. RGS4Δ domain accelerates GTP hydrolysis on Gαi/oGTP, sequestering Gβγ to form Gαβγ heterotrimers^47^. RGS4Δ is tethered to a cryptochrome 2-based CRY2-mCh-RGS4Δ. Upon blue light stimulation, CRY2 dimerizes with its plasma membrane-targeted binding partner, a truncated version of cryptochrome-interacting basic-helix-loop-helix (CIBN)^46^. We expressed α2AR-CFP, CRY2-mCh-RGS4Δ, CIBN-CAAX, and Akt-PH-Venus in RAW264.7 cells. While imaging cells for mCherry and Venus, we exposed a localized membrane region of the cell to blue light (Fig. 5B, blue box). The localized blue light recruited CRY2-mCh-RGS4Δ (Fig. 5B, 1 min). We then activated α2AR globally by adding 100 µM norepinephrine. Cells produced PIP3 only at the opposite side from the CRY2-mCh-RGS4Δ localization (Fig. 5B, 3 and 10 min). Here, the recruitment of RGS4Δ should significantly decrease the lifetime of GαGTP and Gβγ, reducing their concentration to a negligible level at the RGS4Δ localized side of the cell. However, on the opposite side, heterotrimers are activated, Gαi-GTP and Gβγ are generated, where Gβγ stimulates PI3Kγ to induce localized PIP3, orchestrating directional cell migration. Similar to the blue opsin induced (Fig. 5A- Localized), the continuous blue light-directed localized RGS4Δ resulted in attenuation -resistant PIP3 at the opposite edge of the cell (Fig. 5B). Upon termination of blue light, the localized PIP3 gradually disappeared (Fig. 5B, 15 min).

Next, in a cell expressing the same construct combination, we activated α2AR globally first and allowed PIP3 to be produced (Fig. 5C, 3 min) and attenuated (Fig. 5C, 10 min). We then recruited CRY2-mCh-RGS4Δ to one side using localized blue light (Fig. 5C, 15 min, and 20 min). Localized recruitment of RGS4Δ disrupted the PIP3 response attenuation, and the opposite side of the cell showed PIP3 generation. Upon switching CRY2-mCh-RGS4Δ to the opposite side, we were able to switch the side of the PIP3 generation in the same cell (Fig. 5C, 25 min, and 30 min). Here, the data collectively show that, when the GPCR activation is global, the PIP3 attenuation is rapid, while localized GPCR activation, as well as asymmetric G protein activation, deliver sustained and localized PIP3 generations at the GPCR/G protein active site. Data also show that even in a cell with PIP3 attenuation incurred, introducing asymmetry to G protein activation breaks the PIP3 attenuation. Here, we utilized three distinct methods to induce asymmetric signaling. In the first two conditions, signaling asymmetry was introduced before the PIP3 attenuation, i.e. (i) localized GPCR activation through blue light-induced blue opsin activation, where both GPCRs and G proteins remained active, and (ii) global GPCR activation by adding norepinephrine to activate α2AR in a cell with one side of the plasma membrane of the cell with the inhibitor that eliminated activated G proteins. In both conditions, cells showed attenuation -resistant PIP3 generation. In the third condition, i. e. a cell with GPCR activated globally, and PIP3 response is attenuated, G protein activity termination in one side of the plasma membrane rescued the cells from the attenuation. Collectively, this data suggests the involvement of G proteins, likely Gβγ, in the PIP3 attenuation mechanism.

### Sustained localized PIP3 production is facilitated by heterotrimer shuttling to the GPCR active site

**Figure 6:**
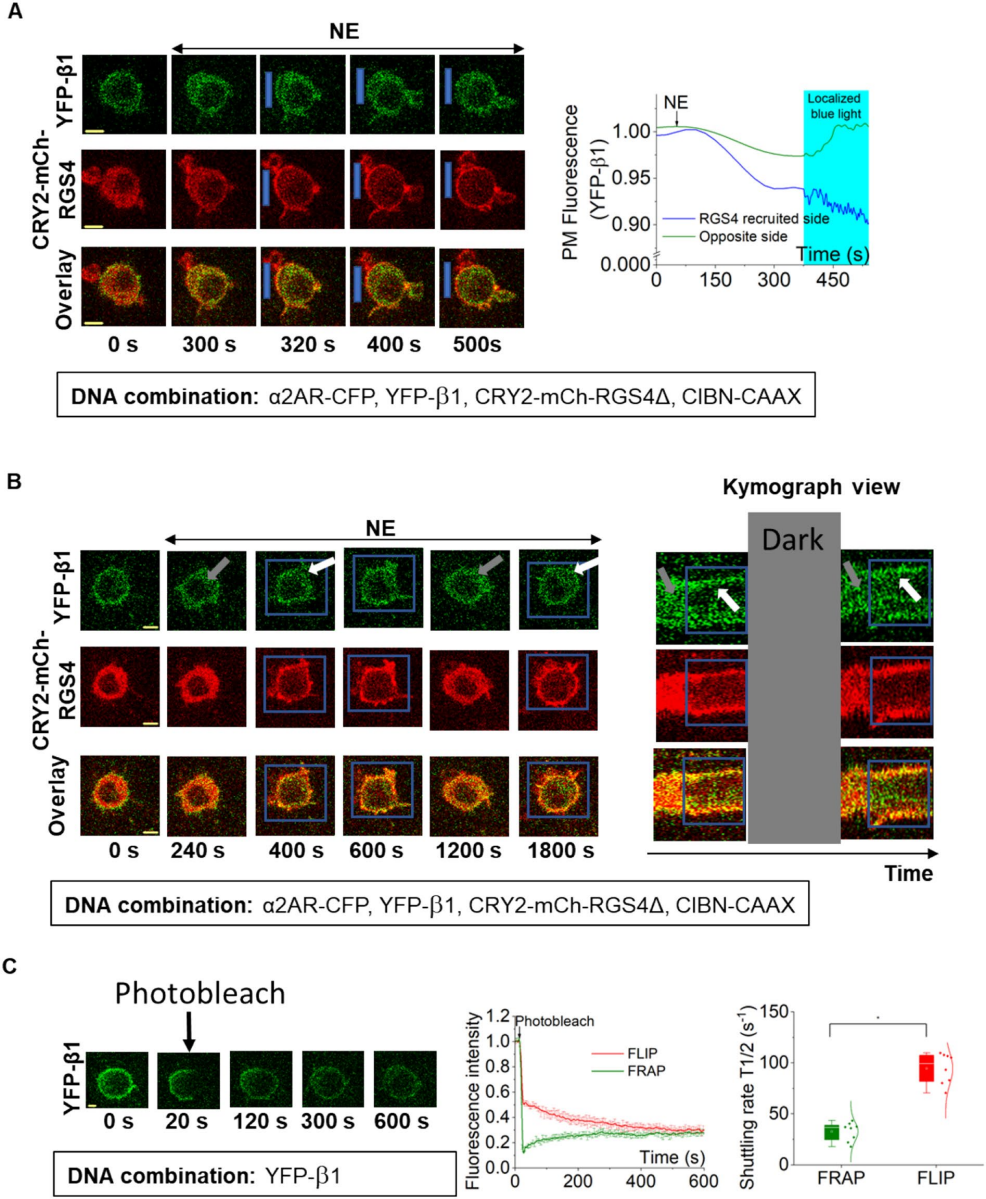
Heterotrimer concentration gradient across the cell breaks PIP3 attenuation. (**A**) Optical targeting of RGS4Δ to one side of a cell induces localized heterotrimer regeneration. RAW264.7 cells expressing α2AR-CFP, YFP-Gβ1, CRY2-mCh-RGS4Δ, and CIBN-CAAX initially exhibited robust Gβ1 translocation upon α2AR activation (300 s). Then, upon localized blue light-induced recruitment of CRY2-mCh-RGS4Δ resulted in an increase of PM-bound YFP fluorescence at the opposite side, suggesting heterotrimer shuttling from the RGS4Δ-recruited side to the opposite side (400 s, 500 s, and green plot). The corresponding plot shows the dynamics of YFP-β1 with localized inhibition of CRY2-mCh-RGS4Δ. (**B**) Reversible blue light targeting of RGS4Δ to the PM allows optogenetic control of heterotrimer concentration at the PM. Upon α2AR activation, cells showed YFP-Gβ1 translocation (240 s, grey arrow). Blue light exposure recruited CRY2-mCh-RGS4Δ to the PM, which induced reverse translocation of Gβ1 to the PM (400 s, white arrow).Termination of blue light that resulted in the release of PM-bound CRY2-mCh-RGS4Δ again resulted in YFP-Gβ1 translocation to the IMs (1200 s, grey arrow). Again, we recruited CRY2-mCh-RGS4Δ to the PM using global blue light and it induced reverse translocation of Gβ1 to the PM (1800s, white arrow). Thus ON–OFF blue light allowed reversible control of heterotrimer concentration. The kymograph shows the dynamics of YFP-β1 with global inhibition by CRY2-mCh-RGS4Δ. (**C**) Fluorescence recovery after subcellular photobleaching (FRAP) in RAW264.7 cells expressing YFP-Gβ1. The corresponding plot shows fluorescence recovery after photobleaching (FRAP) in photobleached PM regions of the cell (green), and fluorescence loss in photobleaching (FLIP) in non-photobleached PM regions. The whisker plot shows the shuttling half times of Gβ1 in FRAP (green) and FLIP (red) (n = 7). Average curves were plotted using cells from ≥ 3 independent experiments. ‘n’ denotes the number of cells’ data used to plot the average curve. The error bars represent SEM (standard error of mean). The scale bar = 5 µm.

Next, we examined molecular reasoning for localized G protein activation to deliver attenuation -resistant PIP3 generation. First, we examined G protein redistribution in a cell where localized RGS4Δ actively reduces the concentration of active G proteins (GαGTP and Gβγ) in one side of a cell in which Gi/o-GPCRs are activated globally. We expressed α2AR, YFP-Gβ1, CRY2-mCh-RGS4Δ, and CIBN-CAAX in RAW264.7 cells. YFP-Gβ1 initially showed a plasma membrane distribution, indicating that it is in the Gαβγ heterotrimer (Fig. 6A, 0 s, and plot). Activation of α2AR induced a gradual Gβ1 translocation to internal membranes (Fig. 6A, 300 s, and plot). Next, we recruited CRY2-mCh-RGS4Δ to one side of the cell using localized blue light (blue box). However, there was no recovery of Gβ1 to the RGS4Δ-recruited side upon blue light exposure (Fig. 6A 320 s, and blue plot). However, we observed an increase of Gβ1 in the plasma membrane at the opposite of the CRY2-mCh-RGS4Δ-recruited side (Fig. 6A 320 s, 400 s,and 500 s, and green plot). In a separate experiment, we activated α2AR with 100 μM norepinephrine, and upon Gβ1 translocation (Fig. 6B, 240 s, and kymograph, grey arrows), we recruited CRY2-mCh-RGS4Δ using global blue light. Demonstrating the GTPase activity of RGS4Δ, cells showed reverse translocation of Gβ1 to the plasma membrane (Fig. 6B, 400 s, 600 s, white arrows, and kymograph). We then kept the cell in the dark for 10 min. Not only CRY2-mCh-RGS4Δ fully returned to the cytosol, we also observed endomembrane localized Gβ1, indicating Gβγ translocation (Fig. 6B, 1200 s, and kymograph). Indicating the reversibility of this process, we again recruited CRY2-mCh-RGS4Δ to the plasma membrane using global blue light and observed reverse translocation of Gβ1 to the plasma membrane (Fig. 6B, 1800s, white arrows, and kymograph). These observations suggest that when RGS4Δ is recruited to the plasma membrane locally, heterotrimers should be formed at the recruited side, while the heterotrimer concentration reaches near zero on the opposite side due to continuing GPCR activity. Therefore, upon localized RGS4Δ recruitment, the cell should have a heterotrimer concentration gradient across the cell. In a concentration gradient, molecules move from higher to lower until an equilibrium is achieved. Therefore, heterotrimers from the RGS4Δ side should shuttle to the opposite side. Since the opposite side heterotrimer concentration is near zero, shuttling should continue indefinitely, as long as global GPCR and local RGS4Δ activities are maintained.

To examine how fast heterotrimers shuttle from one side to the other in a living cell, we expressed YFP-Gβ1 in RAW264.7 cells and photobleached YFP except for a small plasma membrane strip (Fig. 6C, 20 s). We then examined the shuttling of YFP-Gβ1 to the opposite bleached area by calculating Fluorescence loss in photobleaching (FLIP) and Fluorescence recovery after photobleaching (FRAP) at the opposite sides. The shuttling half-time of heterotrimers in FRAP was 32.76 ± 3.25 s, and FLIP was 94.66 ± 5.31 s. When the time duration from RGS4Δ recruitment to the initiation of the PIP3 generation at the opposite side (~ 5 min) is considered, the rate of heterotrimer shuttling is relatively faster . Therefore, it is likely that heterotrimers generated at the RGS4Δ side (as in Figs. 5C, 6A,B) reach the opposite side, and significantly elevate Gβγ concentration compared to the Gβγ concentration in globally GPCR-activated cells (without RGS4Δ recruitment). We propose that this elevated localized Gβγ in asymmetrically G protein-activated cells allow for attenuation-resistant PIP3 generation.

In a globally GPCR-activated cell, at the equilibrium after GPCR activation, heterotrimer concentration at the plasma membrane should go to near zero. Though the Gβγ concentration should go up at the plasma membrane initially, upon translocation, it should be significantly reduced. Since PIP3 generation is Gβγ-dependent, over time PIP3 level at the plasma membrane should significantly reduce, or in other words, attenuated, due to the reduced production. As we showed above (Fig. 5B), in a globally GPCR activated cell with RGS4Δ recruited, the free Gβγ is constantly sequestered in the heterotrimer due to RGS4Δ-induced efficient GαGDP formation, resulting in no PIP3 production.

## Conclusion

**Figure 7:**
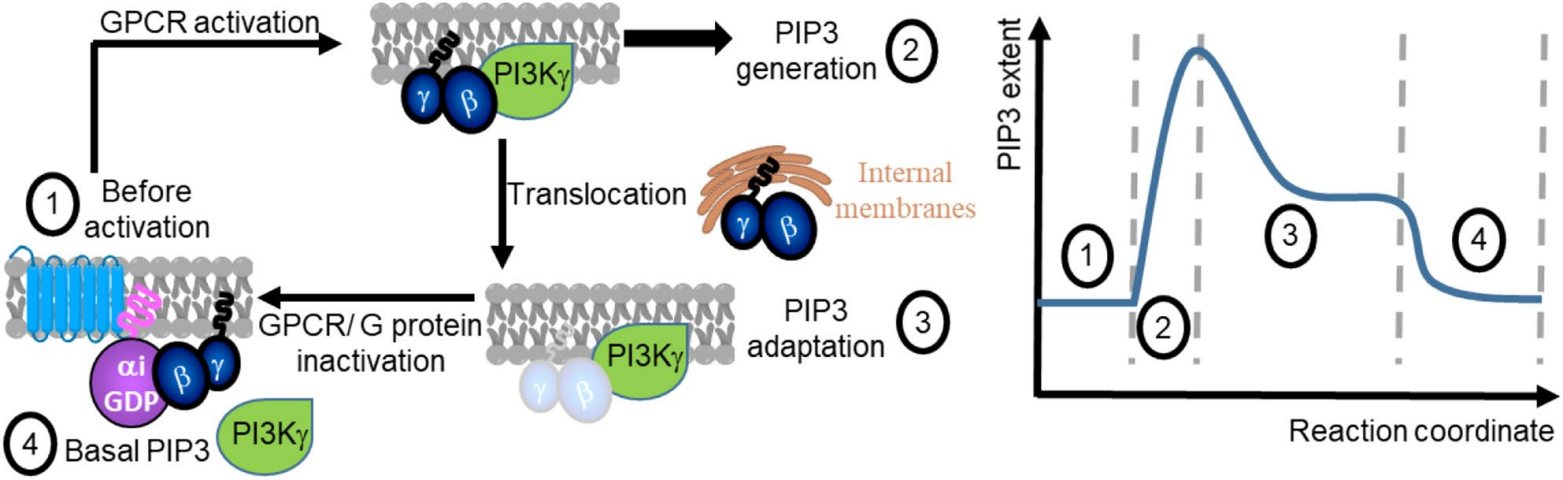
Proposed mechanisms for GPCR-G protein-induced PIP3 regulation. Gi/o-coupled GPCR activation induces robust free Gβγ, which stimulates PI3Kγ resulting in PIP3 generation (1). The subsequent partial PIP3 attenuation (2) is due to the translocation of Gβγ away from the plasma membrane to the internal membranes. The steady state of partial PIP3 attenuation is achieved at the equilibrium of Gβγ translocation between the plasma membrane and the internal membranes. Complete inactivation of GPCR or G proteins result in the PIP3 level returning to the basal level.

Cells and tissues develop signaling adaptation and attenuation mechanisms to avoid overstimulation despite continuous stimuli. Gi-coupled GPCR activation induces a fast-attenuating PIP3 response. Our results indicate that PIP3 attenuation is not due to GPCR desensitization, PIP2 loss at the plasma membrane, or PIP3 degradation by cellular phosphatases.. We show that the attenuation of the GPCR-induced PIP3 response is indeed a form of partial adaptation since the attenuation incurs while continuous receptor stimulation occurs (both the ligand and functional receptors are present). Here, we identified Gβγ translocation from the plasma membrane to endomembranes that control their concentration at the plasma membrane inner leaflet as the underlying mechanisms for the observed PIP3 attenuation (Fig. 7). Since Gβγ recruits PI3Kγ to the plasma membrane where the substrate, PIP2, resides^48^, our data show that plasma membrane-residing-ability or lack thereof of Gβγ regulates PI3K activity—PIP3 generation. Plasma membrane-residence of Gβγ is linked to their membrane affinity, which is governed by the associated Gγ subtype^23^. Our findings further demonstrate that this Gβγ-regulated PIP3 attenuation is Gγ type-dependent. We also decoded the unexpected, attenuation -resistant PIP3 response triggered by the asymmetric GPCR or G protein activation. We showed that the excess heterotrimer availability and the elevated G protein activation at the localized plasma membrane-area, a scenario that is absent when GPCRs-G proteins are globally activated, allows for the sustained localized PIP3. We previously showed that such localized PIP3 sets the migration direction, as well as likely to be involved in neuronal symmetry breaking and differentiation^49^. Further, the ability of cells to produce rapidly attenuating PIP3 upon global GPCR activation should therefore act as a signal for cells to cease the cell-fate-deciding behaviors, including migration, creating autonomous regulation of crucial cell behaviors possible.

Gγ shows distinct cell and tissue type-specific distribution patterns^24^. Therefore, our findings allow for cells with low plasma membrane-affinity Gβγ to show faster and greater PIP3 attenuation while setting the molecular machinery for cells expressing high plasma membrane-affinity Gβγ to have slow-adapting PIP3 responses. Consequently, our newly found regulatory mechanisms in GPCR-G protein-PI3K-PIP3 signaling can allow for knowledge-based signaling predictions on physiological outcomes of different cells and tissues. Since GPCR-G protein signaling is one of the major drug targets and PI3Kγ-PIP3 signaling is implicated in oncogenesis^12,50,51^, our findings will help realize the abundance, impact, and enormity of distinct and diverse cell-tissue-specific signaling regimes of the same pathway, and their implications in health and disease.

## Materials and methods

### Reagents

The reagents used were as follows; Norepinephrine (NE) (Sigma Aldrich), Yohimbine, Bombesin (Tocris Bioscience), PBS, Insulin (Sigma corporation, St Louis, MO), 11-*cis*-retinal (National Eye Institute). Stock solutions of compounds were prepared according to manufacturers' recommendations. Before adding to cells, all stock solutions were diluted in 1% Hank's balanced salt solution (HBSS) or regular cell culture medium.

### DNA constructs and cell lines

DNA constructs used were as follows; DNA constructs used for Blue opsin-mTurquoise, mCherry-Gγ3-CC mutant, fluorescently tagged Akt-PH, CRY2-mCherry-RGS4Δ, and PH, Lyn-HTH have been described previously^8,23,49,52,53^. CIBN-CAAX was cloned into the pcDNA3.1 vector in our lab. NES-Venus-mini-Gi was kindly provided by Professor N. Lambert's laboratory, Augusta University, Augusta, GA. GRPR was a kind gift from the laboratory of Dr. Zhou-Feng Chen at Washington University, St Louis, MO. Fluorescently tagged Gγ3 and Gγ9 subunits, YFP-β1, and αq–CFP were kindly provided by Professor N. Gautam's laboratory, Washington University, St Louis, MO. All constructs were cloned by Gibson assembly cloning (NEB). Cloned cDNA constructs were confirmed by sequencing (Genewiz). Cell lines used were as follows: RAW264.7, CHO, and HeLa cells were purchased from the American Tissue Culture Collection (ATCC).

### Cell culture and transfections

RAW264.7 cells were cultured in Roswell Park Memorial Institute (RPMI) 1640 medium (Corning, Manassas, VA) supplemented with 10% heat-inactivated dialyzed fetal bovine serum (DFBS, Atlanta Biologicals, GA) and 1% penicillin–streptomycin (PS, 10,000 U/ml stock) and grown at 37 °C with 5% CO_2_. Cells were cultured in 35 mm, 60 mm, or 100 mm cell culture dishes (Celltreat). DNA transfections were performed using electroporation. The electroporation solution was prepared with the Nucleofector solution (82 µL), Supplement solution (18 µL), and appropriate volumes of DNA constructs. For each experiment, ~ 2–4 million cells were electroporated using the T020 method of the Nucleofector™ 2b device (Lonza). Immediately after electroporation, cells were mixed with cell culture medium at 37 °C and seeded onto 35 mm cell culture-grade glass-bottomed dishes coated with poly-L-lysine. Cells were imaged ~ 5–6 h post-electroporation.

### Live cell imaging, image analysis, and data processing

The methods, protocols, and parameters for live-cell imaging are adapted from previously published work^42,54,55^. Briefly, live-cell imaging experiments were performed using a spinning disk (Yokogawa CSU-X1, 5000 rpm) XD confocal TIRF imaging system composed of a Nikon Ti-R/B inverted microscope with a 60X, 1.4 NA oil objective and iXon ULTRA 897BVback-illuminated deep-cooled EMCCD camera. Photoactivation and Spatio-temporally controlled light exposure on cells in regions of interest (ROI) were performed using a laser combiner with 40–100 mW solid-state lasers (445, 488, 515, and 594 nm) equipped with Andor® FRAP-PA unit (fluorescence recovery after photobleaching and photoactivation), controlled by Andor iQ 3.1 software (Andor Technologies, Belfast, United Kingdom). Fluorescent sensors such as Akt-PH-mCherry, mCherry-γ3, mCherry-γ9, mCherry-PH, and CRY2-mCherry-RGS4Δ were imaged using 594 nm excitation − 624 nm emission settings; Akt-PH-Venus and Venus-mini-Gi were imaged using 515 nm excitation and 542 nm emission; α2AR-CFP, Gαq-CFP, and Blue opsin-mTq was imaged using 445 nm excitation and 478 nm emission. Before experiments, we selected cells expressing α2AR or blue opsin GPCR by imaging the receptors with the 445 nm laser. In experiments with blue opsin, we imaged cells with blue light before the addition of retinal. However, for experiments with CRY2, instead of using blue light to image the receptors, we used PIP3 production as an indicator of the presence of the receptor. For global and confined optical activation of CRY2 expressing cells, the power of 445 nm solid-state laser was adjusted to 5 mW. Additional adjustments of laser power with 0.1–1% transmittance were achieved using Acousto-optic tunable filters (AOTF). Ophir PD300-UV light meter was used for laser power measurements. Data acquisition, time-lapse image analysis, processing, and statistical analysis were performed as explained previously^54^. Briefly, Time-lapse images were analyzed using Andor iQ 3.1 software by acquiring the mean pixel fluorescence intensity changes of the entire cell or the selected area/regions of interest (ROIs).

### Statistical data analysis

All experiments were repeated multiple times to test the reproducibility of the results. Statistical analysis and data plot generation were done using OriginPro software (OriginLab®). Results were analyzed from multiple cells and represented as mean ± SEM. The exact number of cells used in the analysis is given in respective figure legends. PIP3 generation and attenuation rates were calculated using the Nonlinear Curve Fitting tool (NLFit) in OriginPro. Each plot was fitted to DoseResp (Dose–Response) function under the Pharmacology category in OriginPro. The mean values of hill slopes (P) obtained for each nonlinear curve fitting are presented as mean rates of PIP3 generation or attenuation. PIP3 dynamics plots fit to the Michaelis–Menten function were used to determine the t_1/2_ of PIP3 generation and attenuation. The obtained mean values of Km were taken as the mean t_1/2._ One-way ANOVA statistical tests were performed using OriginPro to determine the statistical significance between two or more populations of signaling responses. Tukey’s mean comparison test was performed at the p < 0.05 significance level for the one-way ANOVA statistical test.

## Supplementary Information

Supplementary Information.

## Data Availability

The datasets used and/or analyzed during the current study are available from the corresponding author on reasonable request.
