# Supplementary material for: The spatial distribution of GPCR and Gβγ activity across a cell dictates PIP3 dynamics
Source: Sci Rep. 2023 Feb 16;13:2771. doi: 10.1038/s41598-023-29639-0 (PMC9935898; doi:10.1038/s41598-023-29639-0)

Supplementary Information

The spatial distribution of GPCR and Gβγ activity across a cell dictates PIP3 dynamics

Dhanushan Wijayaratna[1, 2], Kasun Ratnayake[1], Sithurandi Ubeysinghe[1, 2], Dinesh Kankanamge[1, 3], Mithila Tennakoon[1, 2], Ajith Karunarathne[1, 2]*

[1]Department of Chemistry and Biochemistry, The University of Toledo, Toledo, OH 43606, USA
[2]Department of Chemistry, Saint Louis University, Saint Louis, MO 63103, USA
[3]Department of Anesthesiology, Washington University School of Medicine, Saint Louis, MO 63110, USA

*Corresponding author: wkarunarathne@slu.edu

Figure S1

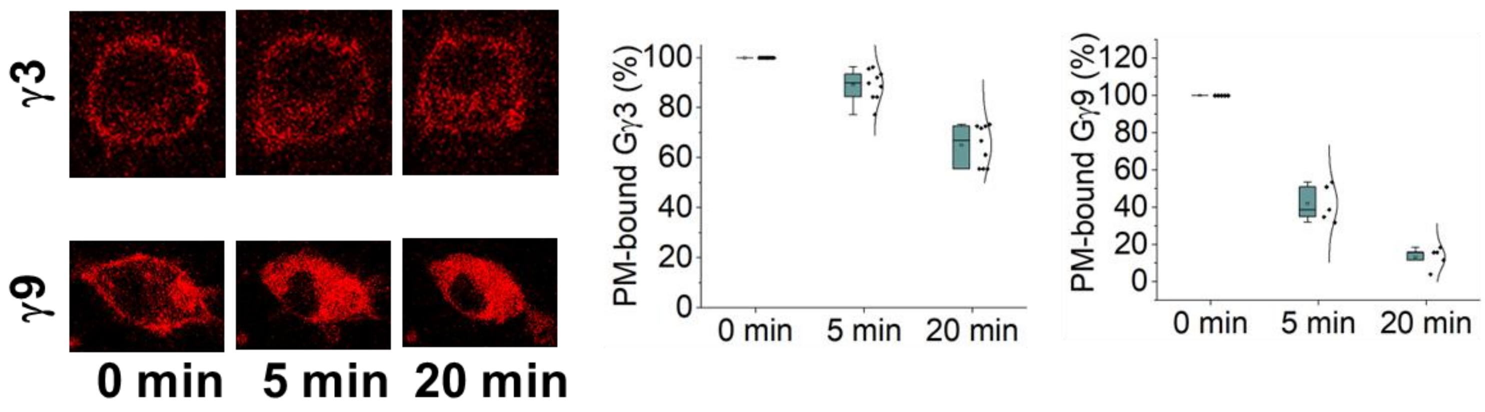

Figure S1. The extent of PM-bound Gγ3 and Gγ9 at 5 minutes and 20 minutes after Gi/o-coupled α2AR activation.

Table S1: Plasma membrane-bound percent Gγ3 and Gγ9 at the steady state of PIP3 adaptation

Gγ type	PM-bound % Gγ (before activation)	PM-bound % Gγ 5 mins after activation	PM-bound % Gγ 20 mins after activation
Gγ3	100 (Overexpression)	89.12048	65.03899
Gγ9	100 (Overexpression)	41.94931	13.11796

Table S2-A: One-way ANOVA statistics for PIP3 generation rates with and without Gq-GPCR activation

Descriptive Statistics- PIP3 production rates				
	N Analysis	Mean	Standard Deviation	SE of Mean
without bombesin	16	0.02858	0.00914	0.00229
with Bombesin	19	0.0111	0.00581	0.00133

Table S2-B

Overall ANOVA- PIP3 production rates					
	DF	Sum of Squares	Mean Square	F Value	Prob>F
Model	1	0.00265	0.00265	47.05388	7.87283E-8
Error	33	0.00186	5.64147E-5		
Total	34	0.00452			

At the 0.05 level, the population means are **significantly** different.

Table S3-A: One-way ANOVA statistics for PIP3 adaptation rates with and without Gq-GPCR activation

Descriptive Statistics- PIP3 adaptation rates				
	N Analysis	Mean	Standard Deviation	SE of Mean
Without bombesin	16	0.00484	0.00199	4.98471E-4
With bombesin	19	0.00385	0.0018	4.13079E-4

Table S3-B

Overall ANOVA- PIP3 adaptation rates					
	DF	Sum of Squares	Mean Square	F Value	Prob>F
Model	1	8.46654E-6	8.46654E-6	2.36795	0.13338
Error	33	1.17991E-4	3.57547E-6		
Total	34	1.26457E-4			

At the 0.05 level, the population means are **not significantly** different.

Table S4-A: One-way ANOVA statistics for PIP3 adaptation extent with and without Gq-GPCR activation

**Descriptive Statistics- PIP3 adaptation extent**				
	N Analysis	Mean	Standard Deviation	SE of Mean
Without Bombesin	16	43.51647	15.62973	3.90743
With bombesin	19	40.24849	17.65828	4.05109

Table S4-B

**Overall ANOVA- PIP3 adaptation extent**					
	DF	Sum of Squares	Mean Square	F Value	Prob>F
Model	1	92.76091	92.76091	0.32997	0.56957
Error	33	9276.99677	281.12111		
Total	34	9369.75768			

At the 0.05 level, the population means are **not significantly** different.

Table S5-A: One-way ANOVA statistics for PIP3 generation rates with and without bpv(phen) inhibitor

**Descriptive Statistics- PIP3 generation rates**				
	N Analysis	Mean	Standard Deviation	SE of Mean
Without bpv(phen)	12	0.02884	0.01193	0.00344
With bpv(phen)	14	0.02589	0.01506	0.00403

Table S5-B

**Overall ANOVA- PIP3 generation rates**					
	DF	Sum of Squares	Mean Square	F Value	Prob>F
Model	1	5.62906E-5	5.62906E-5	0.29921	0.58943
Error	24	0.00452	1.8813E-4		
Total	25	0.00457			

At the 0.05 level, the population means are **not significantly** different.

Table S6-A: One-way ANOVA statistics for PIP3 adaptation rates with and without bpv(phen) inhibitor

**Descriptive Statistics- PIP3 adaptation rates**				
	N Analysis	Mean	Standard Deviation	SE of Mean
Without bpv(phen)	12	0.00382	0.00185	5.33617E-4
With bpv(phen)	14	0.00359	0.00222	5.94051E-4

Table S6-B

**Overall ANOVA- PIP3 adaptation rates**					
	DF	Sum of Squares	Mean Square	F Value	Prob>F
Model	1	3.5036E-7	3.5036E-7	0.08259	0.77629
Error	24	1.01814E-4	4.24225E-6		
Total	25	1.02164E-4			

At the 0.05 level, the population means are **not significantly** different.

Table S7-A: One-way ANOVA statistics for PIP3 adaptation extent with and without bpv(phen) inhibitor

Descriptive Statistics- PIP3 adaptation extent				
	N Analysis	Mean	Standard Deviation	SE of Mean
Without bpv(phen)	12	46.01196	18.5537	5.35599
With bpv(phen)	14	52.79968	15.26877	4.08075

Table S7-B

Overall ANOVA- PIP3 adaptation extent					
	DF	Sum of Squares	Mean Square	F Value	Prob>F
Model	1	297.70369	297.70369	1.04804	0.31617
Error	24	6817.39873	284.05828		
Total	25	7115.10242			

At the 0.05 level, the population means are **not significantly** different.

Table S8-A: One-way ANOVA statistics for PIP3 adaptation extent in Gγ3-CC mutant and Gγ3-WT expressing cells with control cells

**Descriptive Statistics- PIP3 adaptation extent**				
	N Analysis	Mean	Standard Deviation	SE of Mean
Control	13	56.80103	23.85578	6.6164
Gγ3-WT	15	18.72531	7.47989	1.9313
Gγ3-CC	14	6.75863	3.17492	0.84853

Table S8-B

**Overall ANOVA- PIP3 adaptation extent**					
	DF	Sum of Squares	Mean Square	F Value	Prob>F
Model	2	18298.74998	9149.37499	46.08066	5.35237E-11
Error	39	7743.4999	198.55128		
Total	41	26042.24988			

At the 0.05 level, the population means are **significantly** different.

Table S9: Normalized Gγ subtype expression profile in RAW 264.7 cells using RNA seq data

Gγ subtype	Expression (number of reads)	α4A-tubulin expression (number of reads)	Relative Expression (normalized to α4A-tubulin expression)	% expression
Gγ1	0	7404.33	0	0
Gγ2	1444.33	7404.33	0.195066	35.90
Gγ3	4.67	7404.33	0.00063	0.12
Gγ4	1.67	7404.33	0.000225	0.04
Gγ5	560.33	7404.33	0.075676	13.93
Gγ7	42	7404.33	0.005672	1.04
Gγ8	3.33	7404.33	0.00045	0.08
Gγ9	684.67	7404.33	0.092468	17.02
Gγ10	195.33	7404.33	0.026381	4.85
Gγ11	3.67	7404.33	0.000495	0.09
Gγ12	1082.67	7404.33	0.146221	26.91
Gγ13	1	7404.33	0.000135	0.02

Figure S2

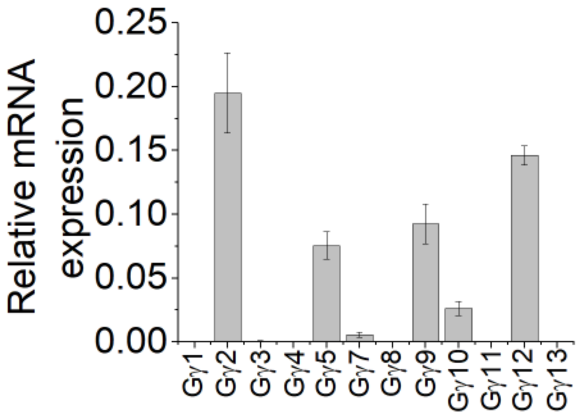

Figure S2. Relative mRNA expression levels of the 12 different Gγ subtypes in RAW264.7 cells. RAW264.7 cells show significant expression of Gγ2, 12, 9, and 5 subtypes. Values were normalized to α4A-tubulin.

Table S10: Normalized expression profile of phosphatases in RAW 264.7 cells using RNA seq data

Phosphatase type	Expression (number of reads)	α4A-tubulin expression (number of reads)	Relative Expression (normalized to α4A-tubulin expression)
PTEN	5484	7404.33	0.740647
Inpp5a	296.33	7404.33	0.040022
Inpp5b	1798	7404.33	0.242831
Inpp5d	6651	7404.33	0.898258
Inpp5e	617.33	7404.33	0.083375
Inpp5f	738.33	7404.33	0.099716
Inpp5k	408.67	7404.33	0.055193
Inpp4a	283	7404.33	0.038221

Figure S3

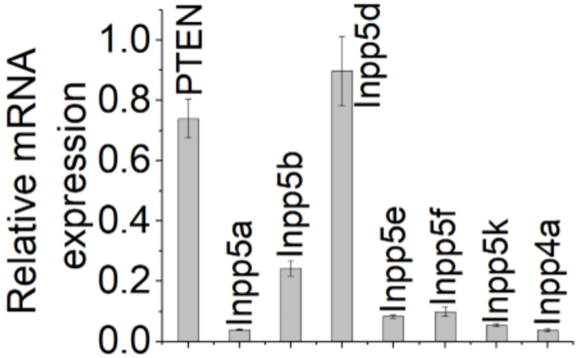

Figure S3. Relative mRNA expression levels of the PIP3 phosphatases in RAW264.7 cells. RAW264.7 cells show significant expression of PTEN and Inpp5d. Values were normalized to α4A-tubulin.

Table S11: Normalized Gαi/o and Gαq/11 expression profile in RAW 264.7 cells using RNA seq data

Gα type	Expression (number of reads)	α4A-tubulin expression (number of reads)	Relative Expression (normalized to α4A-tubulin expression)
Gαi/o	42384.67	7404.33	5.724306
Gαq/11	3902.67	7404.33	0.527079

Figure S4

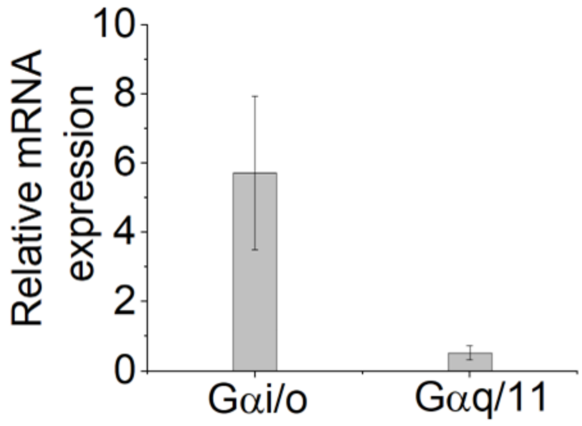

Figure S4. Relative mRNA expression levels of the Gαi/o and Gαq/11 in RAW264.7 cells. RAW264.7 cells show 10-fold higher expression of Gαi/o compared to Gαq/11. Values were normalized to α4A-tubulin.

Figure S5

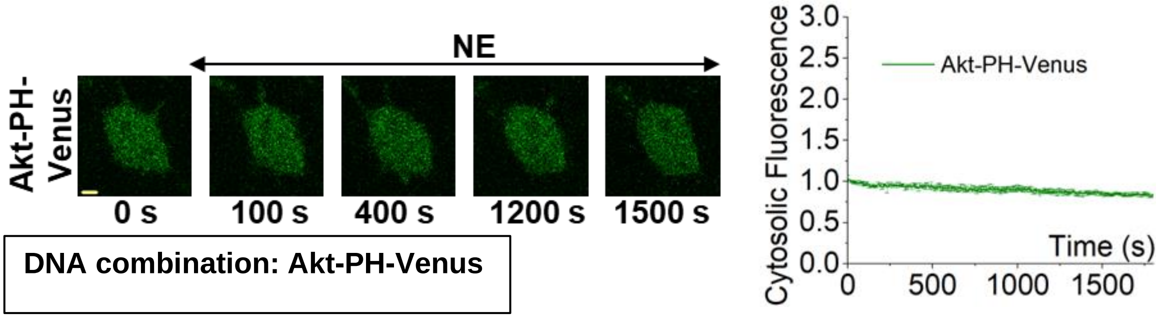

Figure S5. No detectable PIP3 was observed in RAW264.7 cells upon NE addition. 100 µM NE was added to RAW264.7 cells expressing Akt-PH-Venus (PIP3 sensor) at 1 minute. The corresponding plot shows PIP3 sensor dynamics in the cytosol of the cells (n = 10). Average curves were plotted using cells from ≥3 independent experiments. 'n' denotes the number of cells. Error bars represent SEM (standard error). The scale bar = 5 µm.

Figure S6

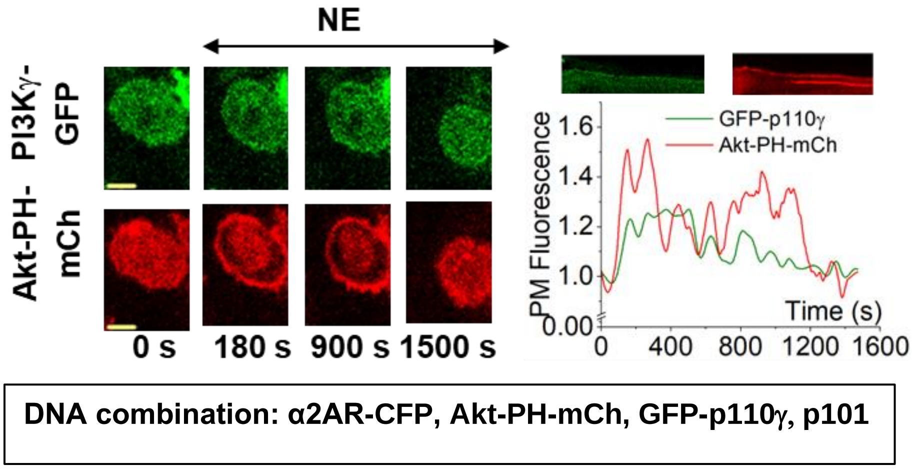

Figure S6. RAW264.7 cells expressing α2AR-CFP, Akt-PH-Venus (PIP3 sensor), and GFP-p110γ, and p101 exhibited transient translocation of GFP-p110γ, and the PIP3 sensor to the plasma membrane from the cytosol upon α2AR activation by 100 µM NE. PI3Kγ (p110γ + p101) transiently translocates to the plasma membrane, which induces PIP3. However, PI3Kγ recovers to the cytosol within a few minutes alongside the attenuation of PIP3. The corresponding kymograph shows GFP-p110γ (green) and PIP3 sensor (red) dynamics in the plasma membrane of the cell given. The scale bar = 5 µm.

Figure S7

Additional cell images

For Figure 1

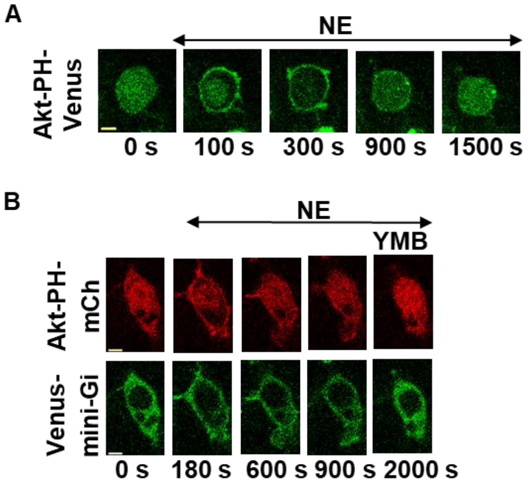

For Figure 2

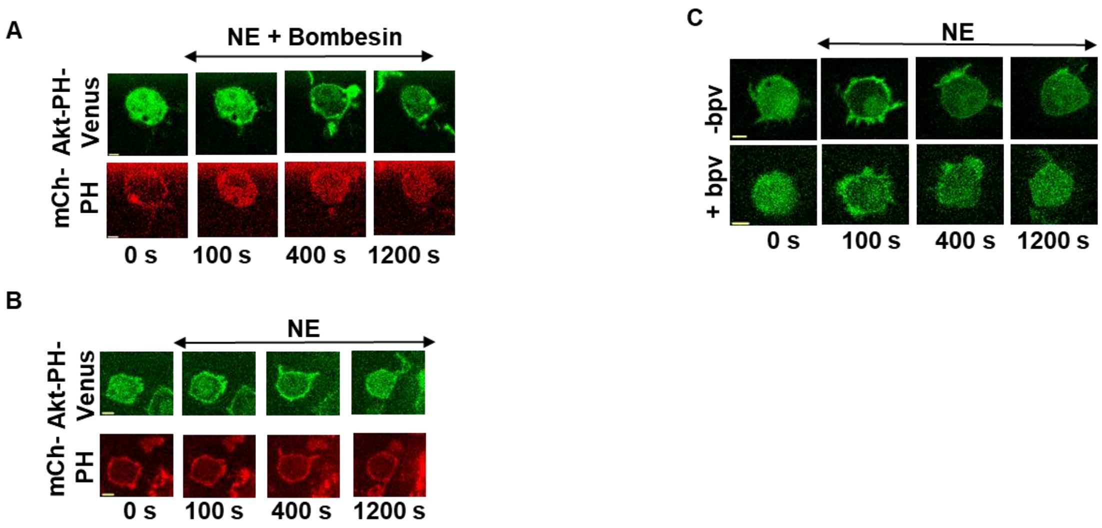

For Figure 3

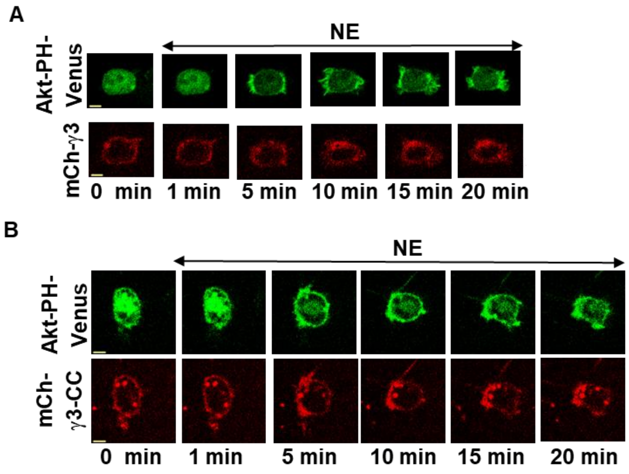

Figure S8

Figure 1 Grayscale images

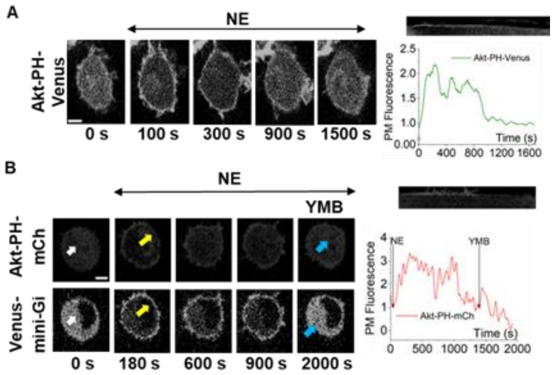

Figure 2 Grayscale images

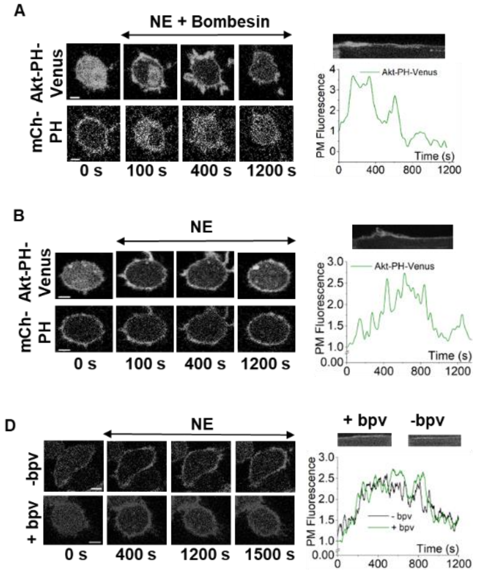

Figure 3 Grayscale images

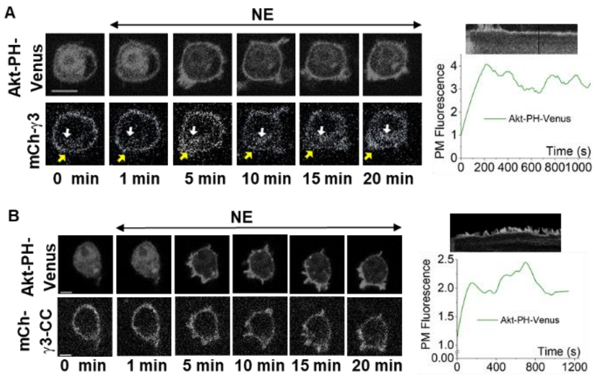

Figure 4 Grayscale images

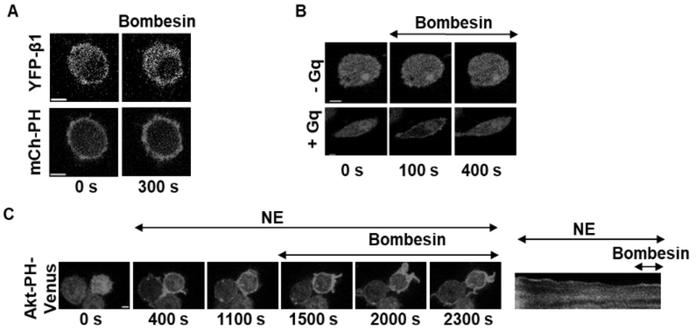

Figure 5 Grayscale images

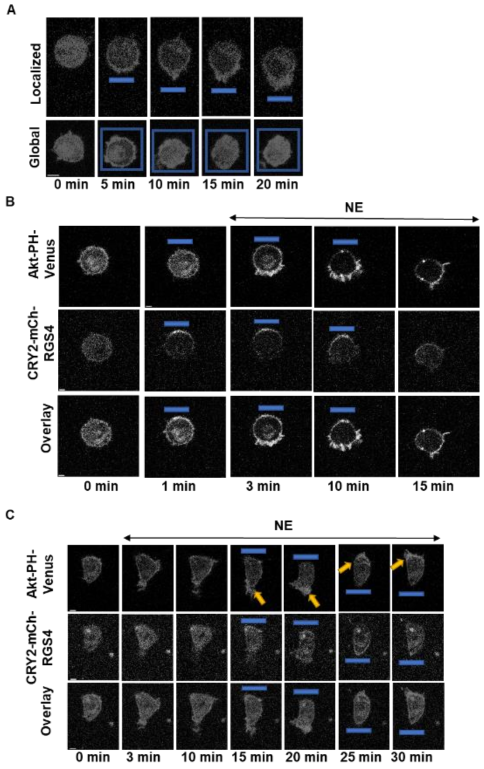

Figure 6 Grayscale images

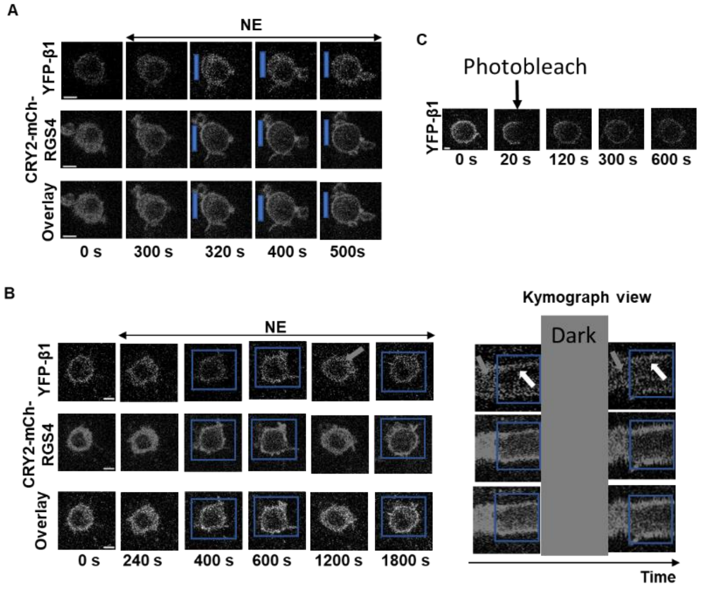

Figure S9

Figure S1 Grayscale images

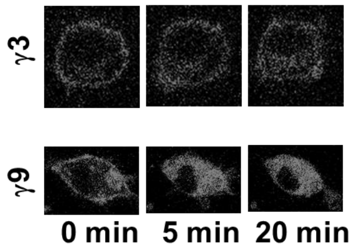

Figure S5 Grayscale images

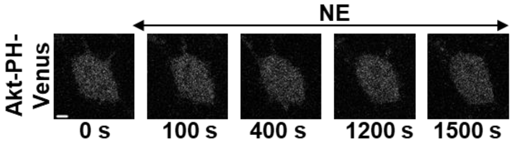

Figure S6 Grayscale images

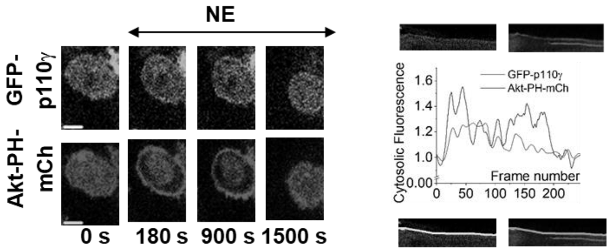

Supplement: Supplementary file 1 — Supplementary Information. [file 41598_2023_29639_MOESM1_ESM.pdf]
